# Magnetic Resonance Imaging as a Biomarker in Diabetic and HIV-Associated Peripheral Neuropathy: A Systematic Review-Based Narrative

**DOI:** 10.3389/fnins.2021.727311

**Published:** 2021-09-21

**Authors:** Matthew C. Evans, Charles Wade, David Hohenschurz-Schmidt, Pete Lally, Albert Ugwudike, Kamal Shah, Neal Bangerter, David J. Sharp, Andrew S. C. Rice

**Affiliations:** ^1^Pain Research, Department of Surgery and Cancer, Faculty of Medicine, Imperial College London, London, United Kingdom; ^2^Department of Brain Sciences, Care Research and Technology Centre, UK Dementia Research Institute, London, United Kingdom; ^3^Royal School of Mines, Imperial College London, London, United Kingdom

**Keywords:** peripheral nerve, magnetic resonance imaging, diabetes, HIV, neuropathy

## Abstract

**Background:** Peripheral neuropathy can be caused by diabetes mellitus and HIV infection, and often leaves patients with treatment-resistant neuropathic pain. To better treat this condition, we need greater understanding of the pathogenesis, as well as objective biomarkers to predict treatment response. Magnetic resonance imaging (MRI) has a firm place as a biomarker for diseases of the central nervous system (CNS), but until recently has had little role for disease of the peripheral nervous system.

**Objectives:** To review the current state-of-the-art of peripheral nerve MRI in diabetic and HIV symmetrical polyneuropathy. We used systematic literature search methods to identify all studies currently published, using this as a basis for a narrative review to discuss major findings in the literature. We also assessed risk of bias, as well as technical aspects of MRI and statistical analysis.

**Methods:** Protocol was pre-registered on NIHR PROSPERO database. MEDLINE, Web of Science and EMBASE databases were searched from 1946 to 15th August 2020 for all studies investigating either diabetic or HIV neuropathy and MRI, focusing exclusively on studies investigating symmetrical polyneuropathy. The NIH quality assessment tool for observational and cross-sectional cohort studies was used for risk of bias assessment.

**Results:** The search resulted in 18 papers eligible for review, 18 for diabetic neuropathy and 0 for HIV neuropathy. Risk of bias assessment demonstrated that studies generally lacked explicit sample size justifications, and some may be underpowered. Whilst most studies made efforts to balance groups for confounding variables (age, gender, BMI, disease duration), there was lack of consistency between studies. Overall, the literature provides convincing evidence that DPN is associated with larger nerve cross sectional area, T2-weighted hyperintense and hypointense lesions, evidence of nerve oedema on Dixon imaging, decreased fractional anisotropy and increased apparent diffusion coefficient compared with controls. Analysis to date is largely restricted to the sciatic nerve or its branches.

**Conclusions:** There is emerging evidence that various structural MR metrics may be useful as biomarkers in diabetic polyneuropathy, and areas for future direction are discussed. Expanding this technique to other forms of peripheral neuropathy, including HIV neuropathy, would be of value.

**Systematic Review Registration:** (identifier: CRD 42020167322) https://www.crd.york.ac.uk/prospero/display_record.php?RecordID=167322.

## Introduction

Diabetic peripheral neuropathy (DPN) is a condition associated with both type 1 (T1DM) and type 2 diabetes mellitus (T2DM), presenting as a distal symmetrical, length-dependent degeneration, usually affecting sensory more than motor nerves. Diabetes mellitus is common, affecting around 8.8% of adults worldwide (International_Diabetes_Federation, 2015), and around half of these are estimated to develop DPN at some point in their lives (Tesfaye, 2011). Neuropathic pain is a common symptom, affecting around a quarter of those with DPN (Van Hecke et al., 2014), and is poorly responsive to analgesic medications (Finnerup et al., 2015).

The pathophysiology of DPN remains incompletely understood, and numerous mechanisms have been proposed, including hyperglycaemia (Won et al., 2012; Xu et al., 2014), increased activity of the polyol pathway (Brownlee, 2001) and dyslipidaemia (Tesfaye et al., 1996) [an excellent review of this topic can be found in Calcutt (2020)]. The polyol pathway received significant attention in the field for some time (Chung, 2003) providing hope that aldose reductase inhibitors (ARIs) could modify disease in DPN. However, meta-analyses of commercially available ARIs to date have not been successful, so these have largely been abandoned in clinical practise (Chalk et al., 2007; Calcutt and Fernyhough, 2016). DPN is classically described as a small fibre neuropathy, with a reduction in intra-epithelial nerve fibre density (IENFD) shown on skin biopsy (Lauria et al., 2010) reflecting a “die-back” pattern of loss of unmyelinated C fibres. However, pathological studies have also shown loss of larger, myelinated fibres, even early in the natural history of the disease (Behse et al., 1977), and autopsy studies of DPN have shown that fascicular damage begins in the proximal sciatic nerve and can be found along the length of the affected nerve (Dyck et al., 1986), which is supported by recent imaging studies showing proximal sciatic involvement in DPN (Groener et al., 2020). The pathological mechanism of the peripheral predominance of symptoms in DPN remains unclear, with likely roles for metabolic dysfunction (Freeman et al., 2016) and neuroinflammation (Leinninger et al., 2004).

HIV infection also causes a symmetrical sensory-predominant polyneuropathy. HIV neuropathy is seen in 20–57% of patients infected with HIV, across low-, middle- and high-income countries (Cherry et al., 2016). The most common form of peripheral nerve lesion found in HIV is a length-dependent axonal degeneration of unmyelinated (C) and thinly myelinated (Aδ) fibres, much like DPN (Cherry et al., 2012b). Two distinct mechanisms appear to be involved in HIV neuropathy: (i) a direct toxic effect of anti-retroviral drugs (ARDs), particularly older di-deoxynucleotide agents (Cherry et al., 2012a; Kamerman et al., 2012), and (ii) an effect of the HIV virus itself, which seems to involve interaction of viral gp120 protein with macrophage CCR5/CXCR4, leading to an inflammatory cascade that results in axonal damage and degeneration (Lee et al., 2003; Moss et al., 2015). Other components of the HIV virus may also be involved in addition to gp120, including viral Transactivator of Transcription (Tat) (Wodarski et al., 2018). The biological mechanisms underlying pain in HIV neuropathy have been poorly elucidated, but various psychosocial factors have been identified (Scott et al., 2018; Scott, 2019).

Treatment of neuropathic pain (and other neuropathic symptoms) can be extremely difficult, with no true disease-modifying therapies for most underlying conditions, and meta-analyses demonstrating that existing therapies have modest efficacy, narrow therapeutic indices and that it is difficult to predict treatment responses at the individual patient level (Finnerup et al., 2015). Part of the challenge of drug development and clinical trial design is the lack of robust biomarkers for neuropathy and neuropathic pain.

Electrophysiological techniques allow probing of the electrical properties of nerves, which help identify axonal and demyelinating processes. However, nerve conduction studies only involve the largest myelinated fibres, so dysfunction of smaller Aδ and C fibres will not be represented. Conversely, whilst skin biopsies can be used to assess epidermal nerve fibre density of the small, peripheral nerve fibres (Smith et al., 2005), this only provides information on structure but not function of nerves, and its application is anatomically limited in practise. The invasive nature of skin biopsy also precludes its use as a longitudinal biomarker. Therefore, we sorely need better, ideally non-invasive biomarkers of peripheral nerve damage, that are easily repeatable for longitudinal study.

Magnetic resonance imaging (MRI) would seem to be the ideal non-invasive biomarker and has found use with various diseases of the central nervous system. However, MRI has had limited application to the study of peripheral nerve disease. Until recently, technological challenges have impeded its use in this field, not of achieving sufficient resolution to provide useful information in structures with cross-sectional areas of 3–50 mm^2^ (i.e., diameter of 2-8 mm). The increased availability of 3 Tesla (T) and 7T systems has changed the landscape in recent years. At higher field strength, MRI can offer fascicular level resolution in larger peripheral nerves (Schmid et al., 2018), offering the possibility of a “virtual biopsy.” MR neurography uses structural *T*_1_-weighted, fat-suppressed *T*_2_-weighted and diffusion weighted sequences to image peripheral nerves. To date, this technology has found use mostly for compressive and traumatic neuropathies (Cudlip et al., 2002), immune mediated sensory and motor neuropathies (Kronlage et al., 2017), and degenerative diseases such as motor neurone disease (MND) (Gerevini et al., 2016).

In this systematic review we review the current state-of-the-art in applying MRI to understanding diabetic and HIV peripheral neuropathies, identifying the main findings in the field, assessing study quality, and suggesting areas for future research. We chose DPN as this is the most common cause of symmetrical polyneuropathy in the Western world. HIV neuropathy was chosen here because its clinical presentation is often indistinguishable from DPN, so would be an appropriate comparison. Also, given that it affects up to 57% of patients with HIV infection it is also not an uncommon condition in the west, but is of particular burden in areas of high HIV prevalence, such as Sub-Saharan Africa.

## Materials and Methods

### Eligibility Criteria

We considered all original research studies relating to MRI in diabetic (T1DM or T2DM) or HIV polyneuropathy. Eligible imaging studies had to involve the peripheral nervous system, rather than the brain or spinal cord. We excluded studies restricted to the dorsal root ganglion, as whilst there is an emerging literature demonstrating pathological changes at this location (Jende et al., 2020c), it has its own unique anatomical and technical challenges for MRI compared with that of imaging of whole peripheral nerves, and therefore we felt warranted its own separate review as the field develops. There was no limit placed on sample size. The only inclusion criterion specific to study-design was that the studies must have had a group of patients with either diabetic neuropathy or HIV neuropathy, as well as a relevant control group (either a group of patients with diabetes or HIV without neuropathy, or healthy controls).

Conversely we excluded isolated case reports; studies with no disease control (i.e., diabetes without neuropathy) and/or healthy control group for comparison; studies investigating other patterns of nerve damage other than symmetrical polyneuropathy (e.g., isolated mononeuropathies, mono-neuritis multiplex, chronic immune demyelinating polyradiculoneuropathy, insulin neuritis, radiculopathy, plexopathy, ganglionopathy); studies involving neuropathies unrelated to the primary conditions of HIV or diabetes (e.g., HIV-related lymphoma, or secondary infections related to HIV or diabetes); and animal studies.

### Information Search/Data Collection

We searched MEDLINE, Web of Science and EMBASE databases from 1946 to 15th August 2020. Search criteria were as follows:

#### Diabetic Neuropathy

(diabetes mellitus/ [MeSH term] OR “diabetes” OR “diabetic”)

AND
(diabetic neuropathies/ [MeSH term] OR polyneuropathies/ [MeSH term] OR small fibre neuropathy/ [MeSH term] OR “neuropathy” OR “neuropathic” OR “neuropathies” OR “polyneuropathy” OR “polyneuropathies”)
AND
(magnetic resonance imaging/ [MeSH term] OR “magnetic resonance imaging” OR “MRI” OR “nuclear magnetic resonance” OR “NMR”).

#### HIV Neuropathy

(HIV/ [MeSH term] OR HIV-1/ [MeSH term] OR HIV-2/ OR “HIV” OR “human immunodeficiency virus”)
AND
(polyneuropathies/ [MeSH term] OR small fibre neuropathy/ [MeSH term] OR “neuropathy” OR “neuropathic” OR “neuropathies” OR “polyneuropathy”)
AND
(magnetic resonance imaging/ [MeSH term] OR “magnetic resonance imaging” OR “MRI” OR “nuclear magnetic resonance” OR “NMR”)

Search results were extracted from each database as RIS files and subsequently imported to Covidence software (www.covidence.org) for analysis, and duplicates were automatically removed. Initial abstract and subsequent full text screening was carried out by two independent reviewers. Any conflicts were resolved by consensus discussion among the reviewers.

### Risk of Bias Assessment

The NIH Quality Assessment Tool for Observational Cohort and Cross-Sectional Studies (http://www.nhlbi.nih.gov/health-pro/guidelines/in-develop/cardiovascular-risk-reduction/tools/cohort) was used. The assessment was performed in duplicate and conflicts were resolved by consensus discussion. The risk of bias tool comprises 14 questions; however 2 of the questions were removed because they were of relevance to prospective cohort studies only, rather than cross-sectional imaging studies forming this review (Question 3: Was the participation of eligible persons at least 50%?; Question 13: Was loss to follow up <20%?). For the remaining questions, “exposure,” or “independent variable” was treated as diabetes or clinical diabetic neuropathy, depending on the study in question. The one exception to this was studies investigating a link between MR changes and specific blood parameters (e.g., troponin T, cholesterol); these were additionally considered as “exposures” where relevant. “Outcome,” or “dependent variable(s)” corresponded to MRI outcomes. Finally, question 14 relates to the control of confounding variables. Based on an a-priori review of the literature for demographic effects on specific MR sequences, we identified 4 variables that should be considered confounds and controlled for accordingly: (i) age; (ii) gender; (iii) disease duration; (iv) BMI (Kollmer et al., 2018; Kronlage et al., 2018, 2019; Groener et al., 2020). This is discussed further in the Discussion section.

### Other Outcome Measures

In addition to the above formal risk of bias assessment, given the relatively small number of studies meeting the inclusion criteria, we appraised each report in the discussion section according to pertinent quality outcomes for MRI studies specifically. These outcomes were: Sample size (whether power calculations have been performed); sample representativeness, and variables controlled for (age, gender, BMI, disease duration, medications, blood glucose and lipid measurements); any aspect of the MRI itself which limits interpretation of studies (field strength, spatial resolution, anatomy scanned); method of nerve segmentation (automated or manual: if automated, whether there is data presented in the paper or elsewhere demonstrating its accuracy; and if manual, whether data is provided on intra- and inter-rater reliability); statistical analysis. Study characteristics are shown in Table 1, and the main results summarised in Table 2.

**Table 1:** Study characteristics.

**References**	**Country conducted**	**Patient group**	**Control group**	**Variables controlled**	**Exclusion criteria**
Griffey et al. (1988)	USA	• Insulin-dependent diabetes with DPN (11) • Insulin-dependent diabetes with DPN taking aldose reductase inhibitor (6)	• Insulin-dependent diabetes without DPN (11) • Non-diabetic healthy control (11)	Nil	• Women • Other causes polyneuropathy ° Renal failure ° Pernicious anaemia ° Alcoholism ° Heavy metal/toxin exposure ° Hypothyroidism
Koechner et al. (1995)	USA	• Diabetes with DPN (13)	• Non-diabetic healthy control (5)	Nil	Nil
Eaton et al. (1996)	USA	• Diabetes with DPN (79)	• Diabetes without DPN (75) • Non-diabetic healthy control (72)	Statistically significant differences in age, gender, BMI., disease duration, and cardiovascular risk factors. No statistical control used.	• Other neuropathy risk factors: ° Renal failure ° Hypothyroid ° Pernicious anaemia ° Alcoholism ° Neurosyphilis ° Heavy metal/toxin exposure
Shibata et al. (1998)	Japan	• Non-insulin-dependent diabetes mellitus (92)	• Non-diabetic healthy control (19)	Age, retinopathy, nephropathy	• Oedema in lower limbs
Pham et al. (2011)	Germany	• T2DM with DPN (10) • T1DM with DPN (2)	• T2DM without DPN (10) • T1DM without DPN (5) • Non-diabetic healthy control (10)	HbA1c, HTN, hyperlipidemia, CHD, myocardial infarction, smoking	Nil
Pham et al. (2015)	Germany	• Diabetes with mild-moderate DPN (25) • Diabetes with severe DPN (10)	• • Diabetes without DPN (15) • Non-diabetic healthy control (25)	Age, sex, disease duration, Retinopathy, Nephropathy, CHD, HTN, Hypertension, Hyperlipidemia, Smoking, HbA1c, Cholesterol, Triglycerides, HDL, LDL, eGFR, BMI	• Age <18 or >75 • Contraindication (CI) to MRI • Symptomatic PAD or CVA • Other neuropathy risk factors ° EtOH excess ° Autoimmune ° Systemic vasculitis ° ESRF
Vaeggemose et al. (2017a)	Denmark	• T1DM with DPN (10)	• T1DM without DPN (10) • Non-diabetic healthy control (10)	Age, Disease duration, BMI, HbA1c	• Severe cardiac/lung disease • Acute or chronic MSK disorder • Lower-limb asymmetric weakness • CI to MRI • Other neuropathy risk factors • Acute metabolic dysregulation • Chronic neurologic diseases • Endocrine disorder
Wu et al. (2017)	China	• Diabetic patients – type not specified (10)	• Non-diabetic healthy control (12)	Sex, age	• Pregnancy • CI to MRI • Hx of leg or knee surgery • Severe cardiac/lung disease • Other neuropathy risk factors ° Metabolic disorder ° Endocrine disorder ° Exposure to neurotoxic agents ° Chronic neurologic diseases ° Neuromuscular/MSK disorder
Felisaz et al. (2017)	Italy	• Diabetes with mild DPN (6) • Diabetes with moderate/severe DPN (10)	• Non-diabetic healthy control (15)	Sex, age	• Unilateral neuropathy • Compressive radiculopathy • Posttraumatic radiculopathy • Other neuropathy risk factors
Vaeggemose et al. (2017b)	Denmark	• Symptomatic mild T1DM Polyneuropathy (13) • Symptomatic severe T1DM Polyneuropathy (11)	• T1DM without DPN (25) • Non-diabetic healthy control (30)	Age, gender, BMI, HbA1c	• Severe cardiac/lung disease • Acute or chronic MSK disorder • Lower-limb asymmetric weakness • Contra-indications to MRI • Other neuropathy risk factors • Acute metabolic dysregulation • Chronic neurologic diseases • Endocrine disorder
Wang et al. (2018)	China	• Diabetes with DPN (22) – 16 T2DM, 6 T1DM	• Diabetes with DPN (20) – 17 T2DM, 3 T1DM • Non-diabetic healthy control (20)	Age, sex, BMI, HbA1c	• Present or past foot osteomyelitis • Present or past foot ulcer • Known hx of foot fracture or surgery • Skin swelling/lesions • Other neuropathy risk factors ° EtOH excess ° Metabolic/toxic factors ° Inflammatory or hereditary
Jende et al. (2018)	Germany	• T2DM with DPN (66) • T1DM with DPN (18)	• T2DM without DPN (19) • T1DM without DPN (17)	Age, BMI, HbA1c, Cholesterol, eGFR, albumin/creatinine ratio	• Age <18 • Pregnancy • CI to MRI • Hx of lumbar surgery • Disc protrusion • Other neuropathy risk factors ° EtOH excess ° Malignancy ° Infection ° Chronic bowel disease ° Hypovitaminosis ° Exposure to neurotoxic agents ° Chronic neurologic diseases ° Micro- or macrocytic anaemia • Monoclonal gammopathy
Jende et al. (2019)	Germany	• T2DM with DPN (64)	• T2DM without DPN (36)	Age, BMI, HbA1c, eGFR	• Age <18 • Pregnancy • CI to MRI • Hx of lumbar surgery • Disc protrusion • Other neuropathy risk factors • EtOH excess • Malignancy • Infection • Hypovitaminosis • Monoclonal gammopathy • Exposure to neurotoxic agents • Chronic neurologic diseases • Renal insufficiency • Microangiopathy
Jende et al. (2020a)	Germany	• Painful DPN (64) – mixture T1DM and T2DM • Non-painful DPN (37) - mixture T1DM and T2DM	• Diabetes without DPN (30) - mixture T1DM and T2DM	Age, sex, HbA1c, diabetes duration, cholesterol, eGFR	• Age <18 • Pregnancy • CI to MRI • Hx of lumbar surgery • Disc protrusion • Other neuropathy risk factors ° EtOH excess ° Malignancy ° Infection ° Hypovitaminosis ° Monoclonal gammopathy ° Exposure to neurotoxic agents ° Chronic neurologic diseases ° Pain disorder other than DPN
Groener et al. (2020)	Germany	• T2DM with DPN (48)	• T2DM without DPN (13) • Non-diabetic healthy control (12)	Age, BMI, HbA1c, diabetes duration, protein:creatinine ratio, eGFR	• Age <18 • Pregnancy • CI to MRI • Other neuropathy risk factors ° EtOH excess ° Malignancy ° Rheumatic autoimmune dx ° Spinal lesions ° Chronic neurologic diseases ° Renal insufficiency
Edward et al. (2020)	Egypt	• T2DM with DPN (30)	• Non-diabetic healthy controls (15)	Age	• Evidence of nerve entrapment • Evidence of other neuropathies: ° Drug-induced/toxic ° Hereditary
Jende et al. (2020b)	Germany	• T2DM with DPN (28)	• T1DM without DPN (23) • Non-diabetic healthy controls (10)	Disease duration, HbA1c, BMI, eGFR	• Age <18 • Pregnancy • CI to MRI • Other neuropathy risk factors • EtOH excess • Malignancy • Infectious diseases • Rheumatic autoimmune dx • Spinal lesions • Chronic neurologic diseases • Renal insufficiency
Vaeggemose et al. (2020)	Denmark	• T2DM with DPN (10)	• T1DM without DPN (10) • Non-diabetic healthy controls (20)	Age, diabetes duration, HbA1c	• Acute metabolic dysregulation • Severe cardiac or lung disease • Musculoskeletal disorders • Other endocrine/neurological disorders • Present or previous asymmetric weakness in the lower limbs • CI to MRI

**Table 2:** Results summary.

**References**	**Magnetic field strength (T)**	**Nerve/Segment**	**Results**				
			**Measurement (spatial resolution)**	Mean ± standard error (^*^ indicates that values have been converted from the original standard deviation to standard error)			***P*-value**
Griffey et al. (1988)	1.5	Sural nerve	**Proton density Dixon sequence, water ratio with CuSO4 phantom (0.312 × 0.615 × 3.0 mm**^**3**^**)** A: Diabetes with “Symptomatic” DPN B: Diabetes with “Treated Symptomatic” DPN C: “Neurologically Asymptomatic Diabetes” D: Non-diabetic healthy control	0.33 ± 0.11 0.26 ± 0.02 0.27 ± 0.11 0.23 ± 0.04			A vs. D: *p* < 0.001 B and C N.S. compared with D
			**Other correlations:** Sural nerve water ratio vs. nerve electrophysiology score Sural nerve water ratio vs. neurological aggregate deficit score				*r* = 0.53, *p* < 0.001 *r* = 0.43, *p* < 0.005
Koechner et al. (1995)	1.5	Sural nerve	**Proton density Dixon sequence, nerve hydration coefficient with CuSO4 phantom (0.312 × 0.615 × 3.0 mm**^**3**^**)** A: Diabetes with “Symptomatic” DPN B: Non-diabetic healthy controls	31.4 ± 2.4 24.6 ± 1.2			No statistical comparison
Eaton et al. (1996)	1.5	Sural nerve	**Proton density Dixon sequence, nerve hydration coefficient with CuSO4 phantom (0.312 × 0.615 × 3.0 mm**^**3**^**)** A: Diabetes with “Symptomatic” DPN B: “Neurologically Asymptomatic Diabetes” C: Non-diabetic healthy control	30.4 ± 5.8% 27.6 ± 5.0% 24.8 ± 3.5%			A vs. C: *p* < 0.05 A vs. B: *p* < 0.05 B vs. C: *p* < 0.05
Shibata et al. (1998)	1.5	Sural nerve	**T**_**1**_**relaxometry, ms (resolution not specified)** A: Non-insulin-dependent diabetes mellitis B: Non-diabetic healthy controls C: Non-insulin-dependent diabetes mellitus: pre-ARI treatment (*N* = 12) D: Non-insulin-dependent diabetes mellitus: post-ARI treatment (*N* = 12)	831 ± 495 472 ± 258 1,056 ± 530 575 ± 335			A vs. B: *p* < 0.001 C vs. D: *p* < 0.001
			Nerve cross-sectional area, mm^**2**^ Non-insulin-dependent diabetes mellitis Non-diabetic healthy controls	2.8 ± 1.8 3.3 ± 1.5			N.S.
			**Other correlations:** T_1_ relaxometry and MNCV T_1_ relaxometry and CV_R−R_ T_1_ relaxometry and FPG T_1_ relaxometry and HbA1c				*r* = −0.426, *p* < 0.001 *r* = −0.295, *p* < 0.001 *r* = 0.350, *p* < 0.001 *r* = 0.337, *p* < 0.001
Pham et al. (2011)	3	Sciatic nerve Tibial nerve Common peroneal nerve (above knee)	**T**_**2**_**-weighted MRI (0.25 × 0.52 × 5.0 mm**^**3**^**)** Number of patients with observable lesions T2DM with DPN T1DM with DPN T2DM without DPN T1DM without DPN Non-diabetic healthy controls	3/10 1/2 0/10 0/10 0/10			No statistical comparison
			Mean contrast ratio between nerve and adjacent muscle A: Diabetic (T1DM/T2DM) with DPN and observable lesions as above (*N* = 4) B: Diabetic control subjects without DPN (*N* = 15) C: Non-diabetic healthy controls (*N* = 10)	4.2 ± 0.9 2.1 ± 0.3 1.9 ± 0.2			A vs. B: p = 0.003 A vs. C: p = 0.004
Pham et al. (2015)	3	Full length of sciatic/tibial/ common peroneal (nerve root to ankle)	**T**_**2**_**-weighted MRI (0.4 × 0.3 × 3.5 mm**^**3**^**)** Number of proximal lesions A: DM with severe DPN B: DM with mild-moderate DPN C: DM without DPN D: Non-diabetic healthy controls Number of distal lesions E: DM with severe DPN F: DM with mild-moderate DPN G: DM without DPN H: Non-diabetic healthy controls Average common peroneal vol per slice (m*m^3^*) I: DM with severe DPN J: DM with mild-moderate DPN K: DM without DPN L: Non-diabetic healthy controls Average tibial vol per slice (m*m^3^*)^†^ I: DM with severe DPN J: DM with mild-moderate DPN K: DM without DPN L: Non-diabetic healthy controls ^†^Divide by slice thickness 3.5 mm to get average cross sectional area (mm^2^)	57 ± 18.4 35 ± 4.0 21 ± 5.5 18 ± 3.6 22 ± 8.1 12 ± 1.8 8 ± 2.9 8 ± 1.4 29.2 ± 3.0 24.6 ± 1.4 23.6 ± 1.2 23.5 ± 1.1 74.4 ± 6.0 62.5 ± 2.7 60.4 ± 3.3 52.8 ± 1.4			A vs. D: *p* < 0.0022 B vs. D: *p* < 0.0005 C vs. D: N.S E vs. E: *p* < 0.0174 F vs. H: N.S G vs. H: N.S. F = 5.61_(3, 71)_, *p* = 0.001 No pairwise comparisons given F = 5.61_(3, 71)_, *p* = 0.001 No pairwise comparisons given
			**Logistic regression by disease group** T_2_ relaxometry Proton density **Regressions/correlations with proton density** Presence of symptomatic DPN NDS NSS Disease duration, HbA1c, BMI, presence of nephropathy/neuropathy, smoking, hyperlipidaemia				N.S. *P* < 0.001 β = 71.25, *p* = 0.032 *r* = 0.3 *p* = 0.009 *r* = 0.27, *p* = 0.02 N.S.
Wu et al. (2017)	3	Tibial nerve Common Peroneal nerve (knee)	**Diffusion tensor imaging (1.25 × 1.28 × 3.0 mm**^**3**^**)** Fractional anisotropy (FA) A: Diabetic neuropathy (10) B: Non-diabetic healthy controls (12) Apparent diffusion coefficient (ADC;**×** *1*0*^3^ mm^2^*/s) A: Diabetic neuropathy B: Non-diabetic healthy controls	**Tibial nerve** 0.534 ± 0.165^*^ 0.593 ± 0.185^*^	**Common peroneal (CP)** 0.553 ± 0.022^*^ 0.623 ± 0.172^*^		FA Tibial: *p* = 0.002 CP: *p* = 0.001
				1.173 ± 0.277^*^ 1.080 ± 0.217^*^	1.128 ± 0.058^*^ 0.993 ± 0.040^*^		ADC Tibial: *p* = 0.001 CP: *p* = 0.009
			**Other correlations** FA vs. motor nerve conduction velocity ADC vs. motor nerve conduction velocity				*r* = 0.460, *p* < 0.05 *r* = −0.479, *p* < 0.05
Vaeggemose et al. (2017a)	3	Sciatic nerve (thigh) Tibial nerve (calf)	**Diffusion tensor imaging (1.36 × 1.36 × 3.0 mm**^**3**^**)** Fractional anisotropy (FA) A: T1DM with DPN B: T1DM without DPN C: Non-diabetic healthy controls Apparent diffusion coefficient (ADC; *x1*0*^3^mm^2^*/s) D: T1DM with DPN E: T1DM without DPN F: Non-diabetic healthy controls **Multi-echo turbo spin echo sequence (0.3 x 0.3 x 3.0 mm)** *T_2_* relaxometry (ms) G: T1DM with DPN H: T1DM without DPN I: Non-diabetic healthy controls Proton density J: T1DM with DPN K: T1DM without DPN L: Non-diabetic healthy controls Nerve cross- *section* al area (m *m^2^*) M: T1DM with DPN N: T1DM without DPN O: Non-diabetic healthy controls	**Sciatic** 0.37 ± 0.02^*^ 0.47 ± 0.03^*^ 0.49 ± 0.01^*^ 1.69 ± 0.08^*^ 1.50 ± 0.02^*^ 1.42 ± 0.04^*^ 86 ± 5.1^*^ 86 ± 3.8^*^ 79 ± 3.2^*^ 314 ± 24.3^*^ 346 ± 18.0^*^ 302 ± 16.8^*^ 29 ± 2.2^*^ 29 ± 2.8^*^ 26 ± 1.6^*^	**Tibial** 0.31 ± 0.02^*^ 0.41 ± 0.02^*^ 0.43 ± 0.03^*^ 1.87 ± 0.14^*^ 1.59 ± 0.06^*^ 1.57 ± 0.08^*^ 65 ± 4.7^*^ 63 ± 3.2^*^ 58 ± 3.8^*^ 429 ± 39.8^*^ 512 ± 35.4^*^ 492 ± 26.6^*^ 8 ± 0.9^*^ 6 ± 0.6^*^ 7 ± 0.6^*^		Sciatic A vs. B: *p* < 0.01 A vs. C: *p* < 0.01 D vs. E: *p* = 0.03 D vs. F: *p* < 0.01 Others: N.S. Tibial A vs. B: *p* < 0.01 A vs. C: *p* < 0.01 Others N.S. All comparisons N.S. All comparisons N.S. All comparisons N.S.
Felisaz et al. (2017)	3	Tibial nerve (ankle)	**IDEAL (Dixon) sequence (0.117 × 0.143 × 2.0 mm**^**3**^**)** Nerve volumes (mm^3^) -NV Fascicles volume (mm^3^) -FV Fascicles to nerve ratio -FNR Cross-sectional areas (mm^2^) -CSA	**Mod-sev DPN (A)** 383.0 ±30.6 251.4 ± 20.3 0.659 ± 0.014 12.97 ± 0.91	**Mild DPN (B)** 326.7 ± 48.4 218.7 ± 29.7 0.677 ± 0.018 12.62 ± 1.27	**Control (C)** 286.8 ± 18.0 198.4 ± 12.8 0.699 ± 0.11 10.22 ± 0.45	NV/FV/FNR A vs. C: p < 0.03 CSA A vs. C *p* < 0.01 B vs. C: *p* < 0.04 Others N.S.
Vaeggemose et al. (2017b)	3	Sciatic nerve (thigh) Tibial (calf) 1	**Diffusion tensor imaging (1.36 × 1.36 × 3.0 mm**^**3**^**)** Fractional anisotropy (FA) A: T1DM with severe DPN (11) B: T1DM with mild-moderate DPN (13) C: T1DM without DPN (25) D: Non-diabetic healthy controls (30) Apparent diffusion coefficient (ADC;**×** *1*0*^3^mm^2^*/s) E: T1DM with severe DPN F: T1DM with mild-moderate DPN G: T1DM without DPN H: Non-diabetic healthy controls **Multi-echo turbo spin echo sequence (0.3 × 0.3 × 3.0 mm**^**3**^**)** Proton density I: T1DM with severe DPN J: T1DM with mild-moderate DPN K: T1DM without DPN L: Non-diabetic healthy controls *T_2_* relaxometry (ms) M: T1DM with severe DPN N: T1DM with mild-moderate DPN O: T1DM without DPN P: Non-diabetic healthy controls Nerve cross-*section*al area (m*m^2^*) Q: T1DM with severe DPN R: T1DM with mild-moderate DPN S: T1DM without DPN T: Non-diabetic healthy controls	**Sciatic** 0.38 ± 0.01^*^ 0.41 ± 0.02^*^ 0.47 ± 0.01^*^ 0.48 ± 0.01^*^ 1.62 ± 0.0.5^*^ 1.62 ± 0.07^*^ 1.52 ± 0.02^*^ 1.47 ± 0.03^*^ 343 ± 23.2^*^ 413 ± 34.4^*^ 403 ± 14.6^*^ 381 ± 14.6^*^ 83 ± 2.1^*^ 82 ± 4.4^*^ 83 ± 1.8^*^ 79 ± 1.5^*^ 28 ± 2.4^*^ 26 ± 1.4^*^ 27 ± 1.6^*^ 21 ± 1.1^*^	**Tibial** 0.31 ± 0.02^*^ 0.34 ± 0.02^*^ 0.41 ± 0.01^*^ 0.42 ± 0.01^*^ 1.78 ± 0.06^*^ 1.74 ± 0.12^*^ 1.59 ± 0.04^*^ 1.52 ± 0.03^*^ 484 ± 26.2^*^ 499 ± 41.3^*^ 570 ± 23.0^*^ 545 ± 20.4^*^ 64 ± 1.8^*^ 63 ± 3.6^*^ 62 ± 1.8^*^ 61 ± 1.8^*^ 8 ± 0.9^*^ 9 ± 1.1^*^ 6 ± 1.4^*^ 7 ± 0.4^*^		A vs. C and D: *p* < 0.01 B vs. C and D: *p* < 0.01 E vs. G and H: *p* < 0.05 F vs. H: *p* < 0.05 Tibial A vs. C and D: *p* < 0.01 B vs. C and D: *p* < 0.01 E vs. G and H: *p* < 0.01 F vs. H: *p* < 0.05 All comparisons N.S. All comparisons N.S. Sciatic T vs. Q, R and S: *p* < 0.01 Tibial S vs. R: *p* < 0.01 T vs. R: *p* < 0.05
Wang et al. (2018)	3	Tibial nerve (ankle)	**T**_**2**_**relaxometry, ms (0.4 × 0.4 × 2.0 mm**^**3**^**)** A: DM with DPN (22) B: DM without DPN (20) C: Non-diabetic healthy control	55.1 ± 4.1 48.9 ± 3.1 45.6 ± 1.9			All comparisons *p* < 0.001
			**Other Correlations:** T_2_ relaxometry vs. HbA1c				*r* = 0.176, N.S
Jende et al. (2018)	3	Sciatic nerve (thigh)	**T**_**2**_**-weighted imaging (0.5 × 0.3 × 4.0 mm**^**3**^**)** A: All neuropathy (T1DM and T2DM) B: All no neuropathy (T1DM and T2DM) C: T1DM with neuropathy D: T2DM with neuropathy E: T1DM without neuropathy F: T2DM without neuropathy	**T**_**2**_**-weighted hypointensities (mm**^**3**^**)** 23.41 ± 2.69 11.43 ± 1.74 19.74 ± 5.57 27.54 ± 3.53 7.52 ± 0.97 16.83 ± 3.16	**T**_**2**_**-weighted hyperintensities (%)** 13.93 ± 0.01 3.18 ± 0.004 19.67 ± 4.13 12.49 ± 1.23 2.80 ± 0.50 2.68 ± 0.43		Hypointensities A vs. B: *p* = 0.002 C vs. D: *p* = 0.046 E vs. F *p* = 0.027 Hyperintensities A vs. B: *p* < 0.0001 C vs. D: *p* = 0.027 Others N.S.
			**Other Correlations:** T_2_-weighted hyperintense lesions vs. tibial compound motor action potential T_2_-weighted hyperintense lesions vs. peroneal nerve conduction T_2_-weighted hyperintense lesions vs. NDS T_2_-weighted hyperintense lesions vs. HbA1c T_2_-weighted hypointense lesions vs. NDS T_2_-weighted hypointense lesions vs. serum triglycerides T_2_-weighted hypointense lesions vs. HDL				*r* = −0.58, *p* < 0.0001 *r* = 0.51, *p* = 0.00002 *r* = 0.52, *p* < 0.0001 *r* = 0.23, *p* = 0.014 *r* = 0.28, *p* = 0.002 *r* = 0.34, *p* = 0.0003 *r* = −0.48, *p* < 0.0001
Jende et al. (2019)	3	Tibial nerve (thigh)	**T**_**2**_**-weighted MRI (0.5 × 0.3 × 4.0 mm**^**3**^**)** Hypointense lipid equivalent lesion (LEL) Maximum length of a lesion, mm Mean cross-sectional area of the tibial nerve (mm^3^)^†^ ^†^Divide by slice thickness 4 mm to get average cross sectional area (mm^2^)	**T2DM with DPN** 1.67 ± 2.03 63.47 ± 2.44 148.20 ± 5.24	**T2DM without DPN** 10.03 ± 0.87 50.07 ± 3.26 122.20 ± 3.82		*p* < 0.001 *p* = 0.001 *p* < 0.001
			**Other Correlations:** Total serum cholesterol vs. lipid equivalent lesion (LEL) load LDL cholesterol vs. LEL load Total serum cholesterol vs. maximum lesion length (MLL) LDL cholesterol vs. MLL Total serum cholesterol vs. mean cross-sectional area (MCA) LDL cholesterol vs. MCA				*r* = −0.41, *p* < 0.001 *r* = −0.33, *p* = 0.003 *r* = −0.44, *p* < 0.001 *r* = 0.38, *p* = 0.001 *r* = −0.38, *p* < 0.001 *r* = 0.33, *p* = 0.002
Jende et al. (2020a)	3	Tibial nerve (thigh)	**T**_**2**_**-weighted MRI (0.3 × 0.3 × 4.0 mm**^**3**^**)** Lesions as % of nerve volume A: Painful DPN B: Non-painful DPN C: Diabetes without DPN Maximum Length of a Lesion D: Painful DPN E: Non-painful DPN F: Diabetes without DPN Cross-*section*al area (m*m^2^*)^†^ G: Painful DPN H: Non-painful DPN I: Diabetes without DPN ^†^Divide by slice thickness 4 mm to get average cross sectional area (mm^2^)	15.15 ± 1.61 10.35 ± 1.66 8.26 ± 1.72 63.62 mm ± 3.01 51.35 mm ± 4.58 41.20 mm ± 4.75 136.4 mm^2^ ± 4.58 144.2 mm^2^ ± 5.80 134.9 mm^2^ ± 6.07			A vs. B: *p* = 0.3 A vs. C: *p* < 0.01 B vs. C: N.S. D vs. E: *p* = 0.0 3 D vs. F: *p* < 0.01 E vs. F: *p* = 0.048 All comparisons N.S.
			**Other Correlations**: Hyperintense nerve lesion load vs. NDS Hyperintense nerve lesion load vs. NSS Hyperintense nerve lesion load vs. tibial nerve conduction velocity Mean nerve cross-sectional area vs. SC level Mean nerve cross-sectional area vs. LDL-C level				*r* = 0.37, *p* < 0.05 *r* = 0.41, *p* < 0.05 *r* = −0.23, *p* < 0.05 *r* = −0.32, *p* < 0.05 *r* = −0.31, *p* < 0.05
Groener et al. (2020)	3	Sciatic nerve bifurcation	**T**_**2**_**-weighted MRI (0.5 × 0.3 × 4.0 mm**^**3**^**)***T_2_*-weighted hyperintense lesions/healthy nerve (%) T2DM with DPN T2DM without DPN Non-diabetic healthy control	8.07 (1–49) 6.13 (3–14) 4.75 (2–12)			All comparisons N.S
			**Other correlations/regressions:** T_2_-weighted hyperintense lesions load vs. sex T_2_-weighted hyperintense lesions load vs. tibial conduction velocity T_2_-weighted hyperintense lesions load vs. tibial nerve amplitude T_2_-weighted hyperintense lesions load vs. QST measure of mechanical detection T_2_-weighted hyperintense lesions load vs. QST measure of mechanical pain T_2_-weighted hyperintense lesions load vs. QST measure of thermal detection / thermal pain				*R*^2^ = 0.674, *p* = 0.31 *r* = −0.362, *p* = 0.005 *r* = −0.276, *p* = 0.035 *r* = −0.312, *p* = 0.007 *r* = 0.246, *p* = 0.036 N.S.
Edward et al. (2020)	1.5	Median nerve (forearm)	**Diffusion tensor imaging (resolution not specified)** Fractional anisotropy (FA) T2DM with DPN Non-diabetic healthy controls Apparent diffusion coefficient (ADC; *× 1*0*^3^mm^2^*/s)T2DM with DPN Non-diabetic healthy controls	**Proximal median** 0.49 ± 0.05 0.51 ± 0.10 1.196 ± 0.199 1.070 ± 0.112	**Distal median** 0.42 ± 0.04 0.46 ± 0.05 1.379 ± 0.209 1.149 ± 0.064		Proximal: N.S Distal: *p* = 0.016 Proximal: *p* = 0.027 Distal: *p* < 0.001
			**Other correlations:** Distal median FA vs. distal radial conduction velocity Distal median FA vs. sensory amplitude Median FA vs. proximal radial conduction velocity Distal median ADC vs. sensory amplitude Distal median FA vs. neuropathy disability score Distal median ADC vs. neuropathy disability score				*r* = 0.299, *p* = 0.02 *r* = 0.257, *p* = 0.048 *r* = −0.267, *p* = 0.039 *r* = −0.278, *p* = 0.032 *r* = −0.518, *p* = 0.003 *r* = 0.482, *p* = 0.007
Jende et al. (2020b)	3	Tibial nerve (thigh)	**Diffusion tensor imaging (1.3 × 1.3 × 4.0 mm**^**3**^**)** Fractional anisotropy (FA) T2DM with DPN T2DM without DPN Non-diabetic healthy controls	0.473 ± 0.056 0.531 ± 0.038 0.549 ± 0.052			ANOVA *p* < 0.001 (no pairwise comparisons)
			**Other correlations:** Tibial FA vs. neuropathy symptoms score (NSS) Tibial FA vs. neuropathy disability score (NDS) Tibial FA vs. tibial nerve conduction velocity^*^ Tibial FA vs. tibial amplitudes^‡^ Tibial FA vs. tibial distal motor latencies^*^ Tibial FA vs. high-sensitivity Troponin T (partial correlation accounting for age and cystatin C levels) ° All T2DM subjects ° T2DM with neuropathy^‡^ Similar data shown for Tibial FA vs. common peroneal electrophysiology (data not shown here)				*r* = −0.36, *p* = 0.009 *r* = −0.52, *p* < 0.001 *r* = 0.37, *p* = 0.011 *r* = 0.57, *p* < 0.001 *r* = −0.32, *p* = 0.029 *r* = −0.31, *p* = 0.030 *r* = −0.61, *p* = 0.001
Vaeggemose et al. (2020)	3	Sciatic nerve (thigh) Tibial nerve (calf)	**Multi-echo turbo spin echo sequence (0.3 × 0.3 × 3.0 mm**^**3**^**)** *T_2_* relaxometry time (ms) A: T2DM with DPN B: T2DM without DPN C: Non-diabetic healthy controls Proton density D: T2DM with DPN E: T2DM without DPN F: Non-diabetic healthy controls **Diffusion tensor imaging (1.36 × 1.36 × 3.0 mm**^**3**^**)** Fractional anisotropy (FA) G: T2DM with DPN H: T2DM without DPN I: Non-diabetic healthy controls Mean diffusivity (MD; **×***1*0*^3^ mm^2^*/s) J: T2DM with DPN K: T2DM without DPN L: Non-diabetic healthy controls Axial diffusivity (AD; **×***1*0*^3^ mm^2^*/s) M: T2DM with DPN N:T2DM without DPN O: Non-diabetic healthy controls Radial diffusivity (RD; **×***1*0*^3^ mm^2^*/s) P: T2DM with DPN Q: T2DM without DPN R: Non-diabetic healthy controls	**Sciatic** 90 ± 5.7^*^ 84 ± 2.5^*^ 81 ± 1.8^*^ 432 ± 25.9^*^ 380 ± 10.8^*^ 370 ± 18.3^*^ − 0.37 ± 0.02^*^− 0.51 ± 0.02^*^ − 0.48 ± 0.01^*^ − 1.75 ± 0.07 − 1.47 ± 0.03^*^ − 1.58 ± 0.04^*^ − 2.42 ± 0.06^*^ − 2.31 ± 0.04^*^ − 2.21 ± 0.04^*^ 1.41 ± 0.07^*^ 1.05 ± 0.03^*^ 1.27 ± 0.04^*^	**Tibial** 78 ± 7.6^*^ 62 ± 3.2 61 ± 2.7^*^ 485 ± 37.3^*^ 548 ± 44.6^*^ 517 ± 24.8^*^ − 0.30 ± 0.02^*^ − 0.45 ^*^ 0.02^*^ − 0.42 ^*^ 0.01^*^ − 1.76 ± 0.08^*^ − 1.48 ± 0.06^*^ − 1.56 ± 0.04^*^ − 2.32 ± 0.07^*^ − 2.19 ± 0.06^*^ − 2.11 ± 0.03^*^ − 1.48 ± 0.09^*^ − 1.13 ± 0.07^*^ − 1.29 ± 0.04^*^		Tibial ANOVA *p* = 0.02 Sciatic ANOVA N.S. All comparisons N.S. Sciatic and tibial: G vs. H p < 0.001 H vs. I *p* < 0.01 Sciatic and tibial: J vs. K *p* < 0.001 J vs. L *p* < 0.05 Sciatic and tibial: M vs. O *p* < 0.01 Sciatic and tibial: P vs. Q *p* < 0.001 Q vs. R *p* < 0.05 Tibial only P vs. R: *p* = 0.01

*For ease of comparison we have also converted any data expressed as standard deviation to standard error, by dividing the standard deviation by square root of N. These data are marked with an*

^*^ *so that readers can refer back to original data if required*.

## Results

### Study Selection

**Figure 1:**
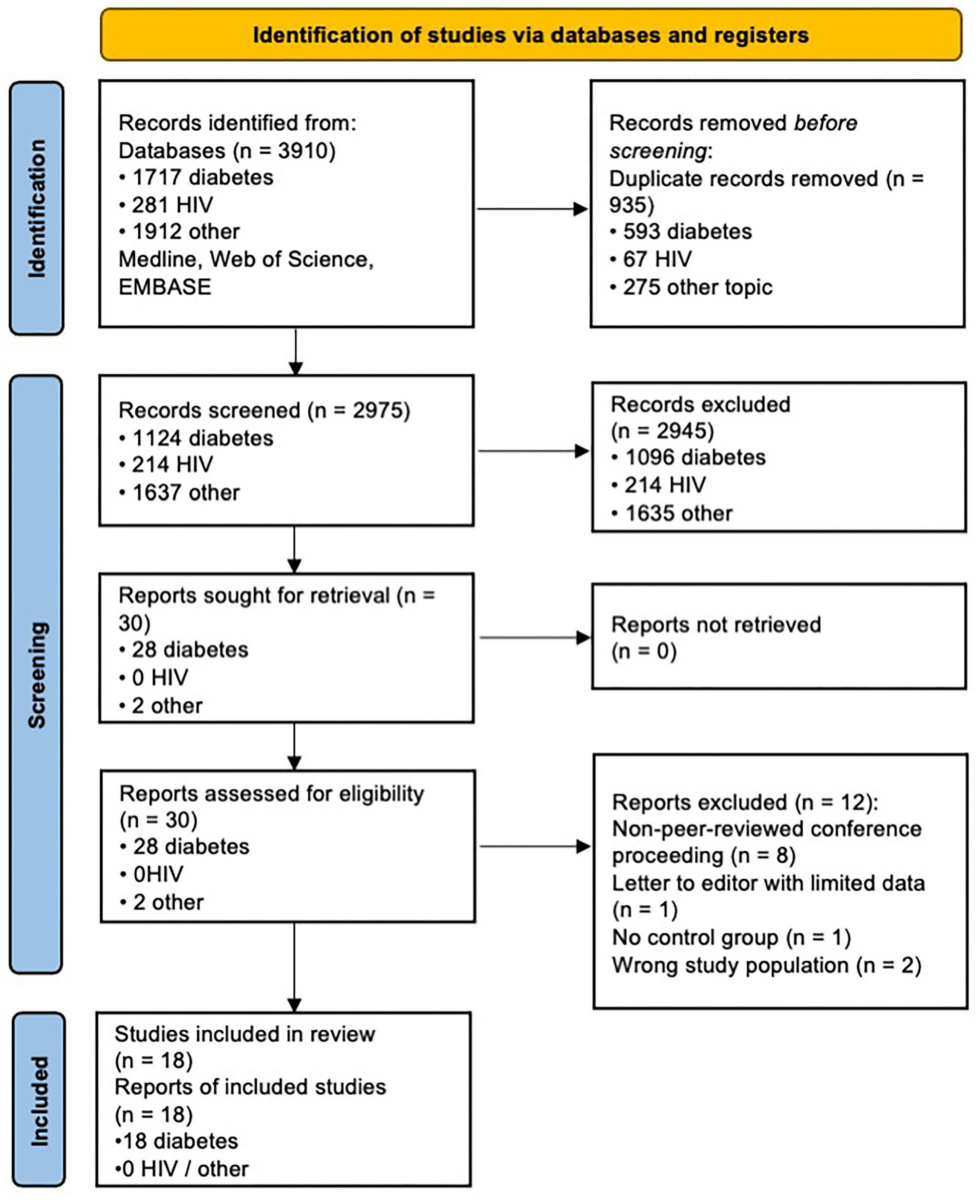
PRISMA chart. Adapted from Page et al. (2021). For more information, visit: http://www.prisma-statement.org/.

Two thousand nine hundred seventy-five papers (duplicates excluded) were screened (by two independent reviewers) by reviewing titles and abstracts according to the inclusion/exclusion criteria itemised in the methods section above, of which 2,945 were deemed irrelevant, leaving 30 papers selected for full text screening. These papers were excluded largely because they involved conditions other than diabetic or HIV neuropathy (1635), or involved diabetic or HIV subjects but were either case reports, or on topics that fell outside of the review (e.g., imaging studies of Charcot arthropathy, or CNS imaging studies of HIV or infective complications of HIV). Twelve studies were excluded from full text screening, either because of study design not meeting inclusion criteria (letter to the editor, case reports, or studies not including any control group), patient population not being appropriate [investigating other forms of neuropathy such as chronic inflammatory demyelination polyneuropathy (CIDP) rather than HIV or diabetic neuropathy], or because the result was a conference proceeding with limited data and with no peer review. Finally, we were left with 18 studies which met eligibility for systematic review (see PRISMA flow chart in Figure 1). All 18 studies were focused on diabetic neuropathy, with no studies found to date investigating HIV neuropathy with MRI. The remainder of the results section will therefore be dedicated to discussing studies of diabetic neuropathy.

### Risk of Bias Assessment

**Figure 2:**
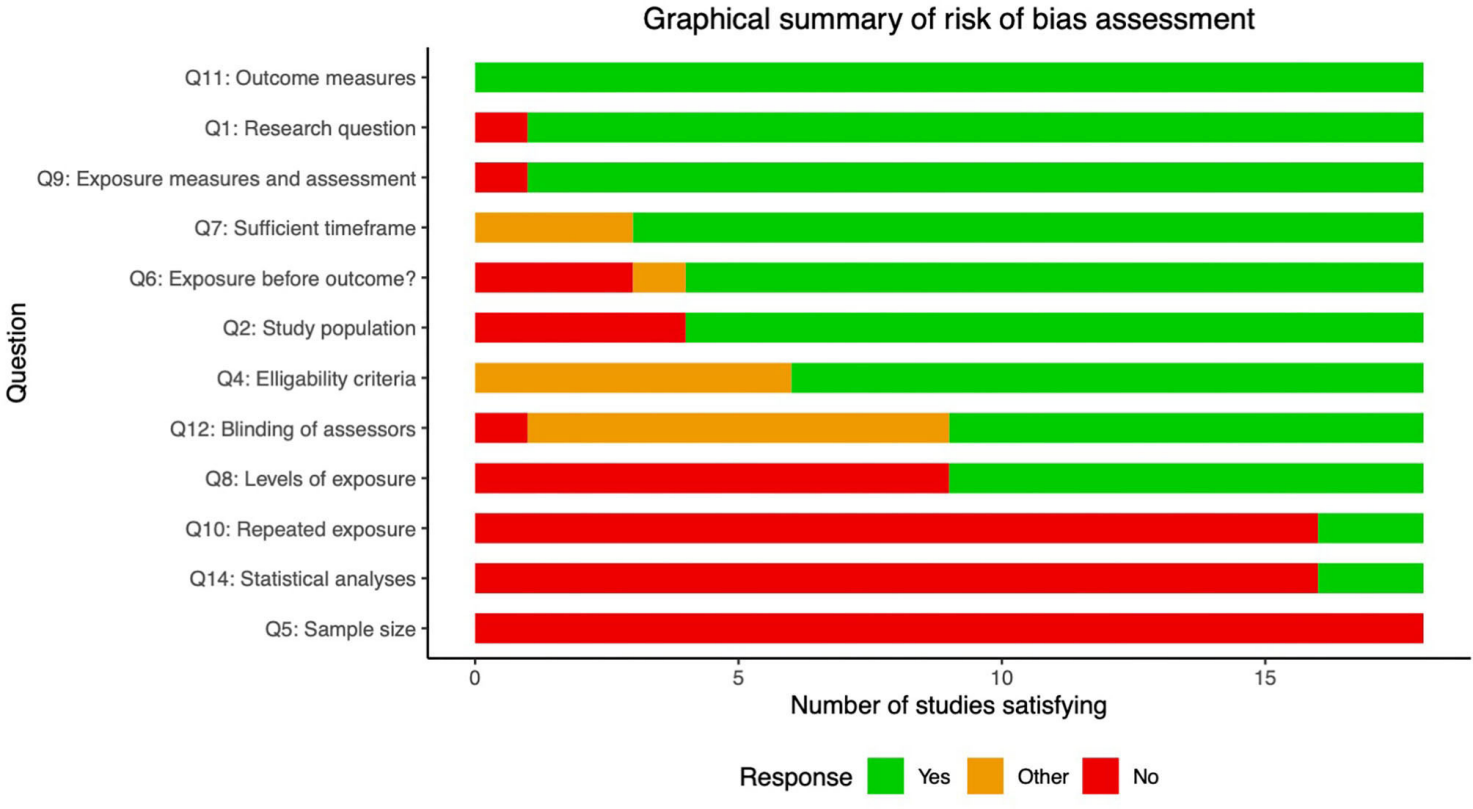
Risk of bias assessment. Summary statistics of risk of bias assessment for the 18 studies included in this review. Descriptions of the relevant questions from the Quality Assessment Tool for Observational Cohort and Cross-Sectional Studies included below. Full explanations of questions and scoring can be found here (https://www.nhlbi.nih.gov/health-topics/study-quality-assessment-tools). Note that questions 3 and 13 were excluded as did not apply to the design used in these studies. 1. Was the research question or objective in this paper clearly stated? 2. Was the Study population clearly specified and defined? 4. Were all the subjects selected or recruited from the same or similar populations (including the same time period)? Were inclusion and exclusion criteria for being in the study prespecified and applied uniformly to all participants? 5. Was a sample size justification, power description, or variance and effect estimates provided? 6. For the analyses in this paper, were the exposure(s) of interest measured prior to the outcome(s) being measured? 7. Was the timeframe sufficient so that one could reasonably expect to see an association between exposure and outcome if it existed? 8. For exposures that can vary in amount or level, did the study examine different levels of the exposure as related to the outcome (e.g., categories of exposure, or exposure measured as continuous variable)? 9. Were the exposure measures (independent variables) clearly defined, valid, reliable, and implemented consistently across all study participants? 10. Was the exposure(s) assessed more than once over time? 11. Were the outcome measures (dependent variables) clearly defined, valid, reliable, and implemented consistently across all study participants? 12. Were the outcome assessors blinded to the exposure status of participants? 13. Were key potential confounding variables measured and adjusted statistically for their impact on the relationship between exposure(s) and outcome(s)?.

The risk of bias assessment is shown for each study in Figure 2. This demonstrates that most (if not all) studies failed to satisfy questions 5 and 14, which relate to sample size justification and confounding variables. For this question we chose the following variables as most pertinent confounds: (i) age; (ii) gender; (iii); BMI; (iv) diabetes duration (if more than one diabetes group in the study).

Question 2 asks whether the populations studied were clearly defined. Many of the earlier studies did not distinguish between type 1 and 2 diabetes, and often did not make clear from the methods section the number of patients from each group, or which patients were on insulin treatment (Koechner et al., 1995; Shibata et al., 1998; Wu et al., 2017; Edward et al., 2020) (14/18 complied). Question 8 asks whether different “levels” of exposure where compared, which in this context relates to whether groups were stratified based on severity or duration of neuropathy or diabetes, to make a more convincing link between disease and MR biomarkers (9/18 complied). Question 10 relates to whether more than 1 time point was used (2/18 complied). Finally, question 12 asks whether outcomes were assessed in a blinded fashion; this was only clearly documented in 9/18 studies. However, of these 7/9 studies used (semi-)automated segmentation of MR images and DTI analysis pipelines which provide minimal opportunity to introduce bias (Pham et al., 2015; Vaeggemose et al., 2017a, 2020; Wu et al., 2017; Jende et al., 2019, 2020a; Edward et al., 2020).

The remaining questions demonstrated high compliance. However, it is worth noting that the three studies by Jende et al. (2018, 2019, 2020a) did not satisfy question 6, which pertains to whether the exposures were measured before the outcomes. In each of these studies, correlational relationships are made between specific MRI values and blood biomarkers (cholesterol and high sensitivity troponin T). As the authors themselves note in the discussion, the cross-sectional nature of the study limits causal relationships being made between variables.

## Narrative Review

### Dixon MRI

The earliest studies attempting to use MRI to non-invasively study peripheral nerves in DPN came from Eaton and Griffey in the late 1980s (Griffey et al., 1988; Koechner et al., 1995; Eaton et al., 1996). In a set of three papers, they used a proton-density-weighted Dixon sequence at 1.5 T as a measure of sural nerve water content. Dixon sequences capitalise on chemical shift, the phenomenon by which protons in fat and water have different resonant frequencies, and therefore over time their corresponding signal components alternate between being in- and opposed-phase. By acquiring two imaging (i.e., 2-point Dixon) or more and combining them mathematically, it is possible to derive water-only and fat-only images from these data. They used water-only Dixon images, normalised to a copper-sulphate (CuSO4) control to derive an estimate of water content from proton density. They demonstrated higher nerve water content in patients with DPN compared with either diabetes without DPN, or healthy controls (Griffey et al., 1988; Koechner et al., 1995; Eaton et al., 1996). In their earlier study, they showed that sural nerve water content positively correlated with deficits on nerve conduction studies. Later, in a larger study using 79 patients with DPN, 75 patients with diabetes without DPN, and 72 non-diabetic healthy controls, Eaton et al. (1996) demonstrated that there was a subset of patients with diabetes without DPN who demonstrated nerve “hyperhydration” (>30% water content of nerve) with nerve conduction values approaching those in the DPN cohort, but when stratifying the DPN patients according to duration of neuropathy, there was a trend towards decreasing nerve water content, despite clearly worsening nerve conduction studies. This led the authors to hypothesise a bell-shaped distribution, with early diabetic neuropathy being associated with an oedematous phase, which “burns out” as the disease progresses.

These studies used a mixed population of T1DM and T2DM (most of which are insulin-dependent), but the proportion of T1DM and T2DM in each group is not clear, which limits interpretation given differences in MRI signature between T1DM and T2DM shown in some later studies. These early studies also did not control for pertinent confounding variables, and demonstrated statistically significant group differences in age, gender, disease duration and cardiovascular risk factors.

It should be noted that the increased water content demonstrated here was reversed with the use of an aldose reductase inhibitor (ARI) (Griffey et al., 1988). As discussed in the introduction, despite the biochemical rationale for why these agents might be effective in DPN, meta-analyses have failed to show any clinically meaningful benefit (Chalk et al., 2007). Therefore, whilst it is possible that use of these agents would have an effect on fluid compartments of nerve tissue, and therefore measures of water content on MRI, it does draw into question the clinical relevance of this imaging biomarker given the lack of clinical efficacy of ARIs.

More recently, however, Felisaz et al. (2017) have used a 3-point Dixon (IDEAL) sequence to obtain ultra-high resolution images of the tibial nerve at the ankle, with the fat-only images best visualising the interfascicular epineurium, and the water-only images (and *T*_1_-weighted turbo spin echo, TSE) giving the best contrast of nerve fascicles. This approach allowed imaging at a very high resolution (0.11 × 0.17 × 2 mm^3^), and segmentation of the nerve fascicles from the surrounding epineurium. The authors were able to demonstrate increased nerve volume and fascicle volume, but also a decreased nerve-to-fascicle ratio in DPN compared with healthy controls, suggestive of expansion of the epineurial connective tissue as well as fascicular enlargement in these patients.

### *T_1_* Relaxometry

Shibata et al. (1998) studied 92 patients with non-insulin-dependent diabetes mellitus compared with 19 non-diabetic healthy controls using *T*_1_ relaxometry (i.e., direct measurement of the longitudinal relaxation time *T*_1_). They found higher *T*_1_ in DPN (831 ± 495 ms) compared with healthy controls (472 ± 258 ms), which the authors proposed was due to nerve oedema. The authors also reported significant positive correlations for *T*_1_ with glycaemic measures and heart rate variability (as a measure of autonomic function), and negative correlations with nerve conduction. Furthermore, they found that in a smaller group of 16 patients, *T*_1_ was reduced by nearly 50% after treatment with Epalrestat, an aldose reductase inhibitor (1,056 ± 530 ms pre-treatment; 573 ± 335 ms post-treatment), although the duration of treatment is not clear from the manuscript. Whilst measures of nerve conduction were carried out as part of this study, it is not clear from the methods how many of the patients with diabetes had a clinical diagnosis of DPN, and so it is not clear to what extent these changes represent neuropathic changes per se, or changes related to diabetes and confounding variables such as BMI. Also as was discussed above, the changes shown in this study were dramatically improved with ARI treatment, which is not thought to be effective for treating DPN.

As described above, Felisaz et al. (2017) also used a *T*_1_-weighted TSE sequence in addition to Dixon imaging to provide ultra-high resolution at 3T of 0.11 × 0.17 mm^2^ in plane with 2 mm slice thickness, allowing separation of the nerve into fascicular and epineurial components, with findings discussed in the previous section.

### *T_2_*-Weighted and Proton Density-Weighted MRI

*T*_2_-weighted imaging-based and *T*_2_ relaxometry are the most commonly used methods for nerve imaging in the literature to date. This has been reported in a number of different ways, the most common using *T*_2_-weighted voxel intensities, with normalisation of signal intensity to some internal (adjacent muscle) or external (healthy control nerve signal intensity) control, and then using arbitrary cut offs to define “hyperintense” or “hypointense” lesions. Pham et al. (2011) was the earliest study to demonstrate proximal *T*_2_ lesions in DPN, initially simply imaging patients with T1DM and T2DM with and without DPN, and had expert neuroradiologists review the *T*_2_-weighted images and code them as having visually apparent lesions or not. They reported that 3/10 of the patients with T2DM and DPN, and 1/2 of the patients with T1DM and DPN had observable lesions, but none of the diabetic patients without DPN (15) or healthy controls (25). They then calculated contrast ratios of *T*_2_-weighted signal intensity in nerve vs. adjacent muscle and showed increased signal intensity in those patients with visually observable lesions, compared with patients with diabetes but no DPN, or healthy controls. The latter is somewhat of an odd statistical comparison, as these subjects have by definition been chosen as having visually higher *T*_2_-weighted signal in segments of the nerve compared with their “control group” and it is not clear how this finding relates to the overall population of patients with DPN as assessed by clinical (rather than radiological) criteria. However, the same group subsequently published a paper with more objective metrics, where they normalised *T*_2_-weighted voxel intensity to age- and sex-matched healthy controls and used a cut off of >1.5x above the average normalised signal intensity to define (hyperintense) lesions. They show both proximally and distally increasing lesion burden from diabetes with no DPN (proximal 21 ± 5.5; distal 8 ± 2.9), to mild-moderate DPN (proximal 35 ± 4.0; distal 12 ± 1.8), to severe DPN (proximal 57 ± 18.4; distal 22 ± 8.1), with significantly more lesions apparent proximally compared with distally. However, the latter statistic does not seem to have been adjusted for nerve cross-sectional area, which would be expected to decrease with more distal location.

These studies only consider hyperintense lesions, but lesions with low *T*_2_-weighted signal intensity are also possible. Jende et al. (2018) took a large cohort of T1DM and T2DM with (64) and without DPN (36). They manually segmented the sciatic nerve in the thigh and defined “hyperintense” lesions as those with signal intensity 25% above adjacent muscle tissue, and “hypointense” as 25% below muscle tissue. Patients with DPN had significant more hyperintense and hypointense lesions compared with those without DPN, and when splitting the DPN group according to diabetes type, there were significant more hyperintense lesions in T1DM and more hypointense lesions in T2DM. Given that hyperintense lesion load correlated with HbA1c, whereas hypointense lesion load correlated with triglycerides and HDL cholesterol, the authors propose that hyperintense lesions are due to complications of hyperglycaemia (such as production of advanced glycation end products, AGEPs), whereas hypointense lesions reflect nerve lipid deposition. Note that the *T*_2_ sequence employed here uses fat suppression, so areas of nerve with lipid deposition would be suppressed causing hypointensities. The fact that areas of low *T*_2_ signal correspond to high *T*_1_ signal is also supportive of the hypothesis that lipid deposition is the cause of *T*_2_ hypointensities in this study. Increased hyperintense and hypointense lesions in DPN compared with diabetic controls have now been replicated in a number of follow up studies (Jende et al., 2019, 2020b; Groener et al., 2020). These data also suggest that taking an average signal intensity as a measure of nerve pathology is fundamentally flawed if there are varying contributions of lesions with high or low signal, as a nerve with equal contributions of both may average to a normal range signal intensity.

Numerous associations have been made between lesions on *T*_2_-weighted imaging and other important outcomes in DPN, including electrophysiological measures (Jende et al., 2018, 2020b; Groener et al., 2020), NDS and NSS scores (Jende et al., 2018, 2020b), quantitative sensory testing (Groener et al., 2020) and glycaemic/lipid measures (Jende et al., 2018, 2019). Jende et al. (2020b) also show that maximum lesion length and lesion load is significantly higher for those with painful vs. painless DPN, and nerve cross-sectional area is correspondingly smaller, suggesting the possibility nerve MRI may be developed as a biomarker for neuropathic pain. Jende et al. (2019) also followed up their finding of an association of *T*_2_-weighted hypointense lesions with dyslipidaemia in a follow up study focusing on T2DM only. Somewhat in contrast to their previous findings in a mixed sample of patients with diabetes, they demonstrated a negative correlation between hypointense lesion load and total (*r* = −0.41), HDL (*r* = −0.30) and LDL cholesterol (*r* = −0.33), suggesting that excessive lowering of cholesterol with statin use might actually promote DPN. This is in line with evidence that statins may limit the supply of cholesterol required for nerve repair (Gaist et al., 2002; Novak et al., 2015) and demonstrates the value of MRI biomarkers in informing pathogenesis. However, caution should be taken in interpreting this cross-sectional study, especially in light of the clear benefit of cholesterol lowering for macrovascular complications in diabetes (Hebert et al., 1997). Longitudinal and randomised control designs would be of benefit to better understand this relationship.

### *T_2_* Relaxometry

Signal intensity in *T*_2_-weighted images is largely determined by the transverse relaxation time (*T*2) of the tissue and its proton density, and some studies have calculated these more direct measures to study pathology in DPN. Vaeggemose et al. (2017a) calculated *T*_2_ in patients with T1DM, and whilst they did not report significant group differences, there was a trend to longer *T*_2_ for DPN in the sciatic nerve (DPN 86 ± 5.1 ms; control 79 ± 3.2 ms) and distal tibial nerve (DPN 65 ± 4.7 ms; control 58 ± 3.8 ms). The same group have reported this metric in two follow up papers (Vaeggemose et al., 2017b, 2020), and most recently Vaeggemose et al. (2017b) reported statistically longer *T*_2_ in the distal tibial nerve for DPN (78 ± 7.6 ms) compared with diabetes without DPN (62 ± 3.2 ms) and healthy controls (61 ± 2.8 ms), with a non-significant trend in the same direction for the sciatic nerve. It should be noted that the sample sizes in the studies by Vaeggemose et al. are relatively modest with around 10 subjects per group. Wang et al. (2018) used a similar technique in a mixed group of T1DM and T2DM with a larger sample size and showed progressively longer *T*_2_ in healthy controls (45.6 ± 1.9 ms) compared to diabetic patients without DPN (48.9 ± 3.1 ms) and those with DPN (55.1 ± 4.1 ms), with no relationship between *T*_2_ and HbA1c. In contrast to these findings, Pham et al. (2015) used logistic regression to investigate whether there was a significant difference between patients with DPN of different severities and healthy controls and found a significant effect of proton density (severe DPN 360 ± 22.9; controls 288 ± 13.4), but no effect of *T*_2_ relaxation. They also showed small but significant correlations between proton density and scores on the neuropathy disability score (NDS) and neuropathy symptom score (NSS). The authors propose that the findings discussed above relating to elevated signal in *T*_2_-weighted imaging actually relate to changes in proton density rather than *T*_2_ itself, and that this suggests a change to the macromolecular environment through, for example, production of AGEPs, rather than due to tissue oedema. However, this is not born out by other studies which have failed to show an effect of proton density (Vaeggemose et al., 2017a,b; Vaeggemose et al., 2020), although the smaller sample size of these studies should be taken into consideration.

### Diffusion Tensor Imaging

Diffusion tensor imaging provides various metrics that can reflect aspects of nerve integrity, including fractional anisotropy (FA), apparent diffusion coefficient/mean diffusivity (ADC/MD), radial diffusivity (RD) and axial diffusivity (AD). FA is a measure of the directionality of proton diffusion, with 0 representing isotropic diffusion and values closer to 1 indicating a strongly preferred direction of diffusion. Due to water diffusing preferentially along the axis of axons, nerves demonstrate higher FA values, and a reduction indicates loss of nerve structural integrity. Studies have consistently shown a reduction in FA in patients with DPN compared with both healthy controls and diabetic patients without DPN, for the sciatic nerve (Vaeggemose et al., 2017a,b; Vaeggemose et al., 2020), tibial nerve (Vaeggemose et al., 2017a,b; Wu et al., 2017; Vaeggemose et al., 2020; Jende et al., 2020a), and common peroneal nerve (Wu et al., 2017), which has been demonstrated at the thigh and ankle level. Edward et al. (2020) also demonstrated reduced FA in the distal median nerve in the wrist. ADC or MD is a measure of the average diffusivity of protons across all directions, with higher values in nerves suggesting some degree of axonal disruption (Tievsky et al., 1999). In line with the decreased FA values, the above studies also demonstrate increased MD values in each of the nerves (Vaeggemose et al., 2017a,b, 2020; Wu et al., 2017; Edward et al., 2020; Jende et al., 2020a). Importantly there seems to be clear evidence of increased MD and decreased FA in DPN compared with diabetes without DPN as well as healthy controls (Vaeggemose et al., 2017a,b; Jende et al., 2020a), and worsening of values in line with severity of DPN (Vaeggemose et al., 2017b). Therefore, these changes seem to reflect neuropathic changes per se, rather than changes related to diabetes.

Other important metrics can also be extracted from the diffusion tensor. AD represents the maximum diffusivity of protons along any axis (i.e., along the nerve axon), whereas RD is a measure of diffusivity perpendicular to this. Early work suggested that AD is sensitive to axonal loss and RD to demyelination, but it is now thought to be more complex than this, with RD also affected by demyelination, axon loss or reduced axonal density. One study to date has investigated these parameters in DPN, showing generally higher AD, RD and MD in DPN compared with diabetes without DPN, and higher for diabetes without DPN compared with healthy controls (Vaeggemose et al., 2020). However, the authors were less consistently able to show a significant difference between DPN and healthy controls, in part due to the increased variance in these measures in the DPN group and may be a reflection of the relatively small sample size in the diabetes groups (10 per group).

### Nerve Cross-Sectional Area

Cross sectional area studied has most commonly been studied in the sciatic nerve and its branches at the level of the thigh. Note that some studies report true cross-sectional areas (Shibata et al., 1998; Vaeggemose et al., 2017a,b, 2020), whilst others report slice volume (cross-sectional area x slice thickness) (Pham et al., 2015; Jende et al., 2019, 2020b). The slice volumes discussed below have been converted to areas by dividing by slice thickness in order to allow for comparison between studies.

Two studies by Vaeggemose et al. (2017a,b) reported CSA for the sciatic nerve (encompassing both tibial and peroneal components) at the level of the distal thigh. In an earlier study (Vaeggemose et al., 2017a) there was a higher CSA for T1DM with (29 ± 2.2 mm^2^) and without DPN (29 ± 2.8 mm^2^) compared with non-diabetic healthy controls (26 ± 1.6 mm^2^), but this was not statistically significant. However, the sample size was only 10 per group in this study so may not have been powered for this comparison. The authors repeated this metric in a larger sample (Vaeggemose et al., 2017b) and showed statistically higher CSA in all diabetic patients (28 ± 2.4 mm^2^ for severe DPN, 26 ± 1.4 mm^2^ in mild DPN, and 27 ± 1.6 mm^2^ in T1DM without DPN) compared with healthy controls (21 ± 1.1 mm^2^). Jende et al. (2019) also scanned the sciatic nerve at the distal thigh, but restricted analysis to the tibial component. They did not include a healthy control group but showed an increased CSA for patients with T2DM with DPN (37.1 ± 1.3 mm^2^) compared with those without DPN (30.6 ± 0.9 mm^2^). In a follow up study, the same group compared a mixed population of T1DM and T2DM with painful neuropathy (34.1 ± 1.1 mm^2^), painless neuropathy (36.1 ± 1.5 mm^2^) and no neuropathy (33.7 ± 1.51 mm^2^), demonstrating no statistically significant group differences (Jende et al., 2020b). However, the authors did demonstrate a negative correlation between CSA and conduction velocities of the tibial and common peroneal nerves, suggesting a relationship between CSA and nerve function, but not painful symptoms.

Pham et al. (2015) scanned the tibial and peroneal nerves of both the thigh and lower leg, and report average cross-sectional areas across the whole length of the nerve in DPN. They showed a significant group difference with ANOVA for both the tibial and common peroneal nerves. Whilst they did not report pairwise comparisons, for the tibial nerve there seems to be a stepwise increase in CSA compared to healthy controls (15.1 ± 0.4 mm^2^) for diabetes without DPN (17.3 ± 0.9 mm^2^), mild-moderate DPN (17.9 ± 0.8 mm^2^) and most prominently, severe DPN (21.3 ± 1.7 mm^2^). However, for the common peroneal nerve there only seems to be an increase from healthy controls (6.7 ± 0.3 mm^2^) for the severe DPN group (8.3 ± 0.9 mm^2^).

Finally, of studies investigating the tibial nerve CSA in the lower leg, Vaeggemose et al. (2017b) showed a higher CSA for patients with moderate DPN (9 ± 1.1 mm^2^) compared with both T1DM without DPN (6 ± 1.4 mm^2^) and healthy controls (7 ± 0.4 mm^2^), but this was not replicated in another study by the same group (Vaeggemose et al., 2017a). Felisaz et al. (2017) investigated distal tibial nerve structure in diabetic neuropathy using ultra-high resolution MRMRI and showed higher CSA in mild DPN (12.6 ± 1.3 mm^2^) and moderate-severe DPN (13.0 ± 0.9 mm^2^) compared with non-diabetic healthy controls (10.2 ± 0.5 mm^2^). Interestingly, this study obtained sufficient resolution to segment out the fascicles from surrounding epineurium and found a decreased fascicle-to-nerve ratio, most noticeable for severe DPN, suggestive of some expansion of the interfascicular epineurium as well as enlargement of the fascicles themselves. Only one study has reported sural nerve CSA in diabetes (Griffey et al., 1988), and there was no significant difference between groups noted. However, this study was published over 20 years ago using a 1.5 T system, so was likely limited by available signal-to-noise ratio.

Taken together, there seems to be reasonable evidence that diabetes (type 1 and type 2) increases the cross-sectional area of nerves (whether or not neuropathy is present), but there is insufficient evidence to suggest that nerve CSA is higher in patients with DPN compared with diabetic patients without DPN, or that it tracks with severity of DPN or painful neuropathy. The values reported here for CSA of the lower extremity nerves are largely in line with the ultrasound literature with regards to healthy controls (Lee and Dauphinée, 2005; Cartwright et al., 2008; Seok et al., 2014), save for the Vaeggemose study which seem much lower (26 mm^2^) than values reported for the sciatic nerve at the thigh in healthy individuals (42–52 mm^2^). The data are also broadly in line with the ultrasound literature on DPN which shows that nerves are larger in diabetes compared with non-diabetic nerves and are larger in those with less well-controlled disease, including in those with DPN (Riazi et al., 2012; Breiner et al., 2017).

## Discussion

There is increasing interest in using MRI as a non-invasive biomarker in various neuropathies, including DPN. In this review we use systematic search tools to summarise this emerging field, with discussing biomarkers using *T*_1_-weighted, *T*_2_-weighted, proton density-weighted, relaxometry and diffusion tensor imaging. We also highlight the lack of research in HIV neuropathy. We will now discuss caveats and challenges in this field, and potential for future developments.

### Confounding Variables and Control Groups

Choosing which confounding variables to control for with peripheral nerve imaging is difficult, as there is scarcity of evidence for how MRI metrics change with age and other demographic factors for peripheral nerve imaging compared with more commonly used imaging modalities like brain imaging. However, one study from Bendszus et al. used MR neuropathy in 60 healthy volunteers to examine the relationship with various demographic variables. For nerve CSA they found a trend towards increasing size with age, which was more pronounced at the sciatic nerve in the thigh (Kronlage et al., 2019). This finding is in agreement with some previous ultrasonographic studies showing increasing size with age (Cartwright et al., 2008). They also found a positive correlation between cross-sectional area and weight, height and BMI (Kronlage et al., 2019). *T*_2_ was not found to be associated with any demographic variable in this study, and proton density was strongly negatively correlated with weight and BMI. Proton density also had a negative correlation with age in the sciatic nerve at the thigh, possibly secondary to fatty infiltration.

In this study, gender was only moderately associated with nerve CSA, but this relationship disappeared when accounting for bodyweight. In a second study, the same group investigated demographic influences in diffusion tensor parameters (Kronlage et al., 2018), showing an age-related decrease in FA, caused by a reduction in axial, and an increase in radial diffusivity. FA was also negatively correlated with height, weight and BMI, and there was no association with gender for any metric after accounting for bodyweight. Whilst these studies on normal controls do not seem to show evidence for an effect of gender, one MR neurography study in DPN showed that male sex was associated with higher *T*_2_-weighted lesion volume in subjects with diabetic neuropathy (Groener et al., 2020). Age has also been associated with changes in other MR parameters including magnetisation transfer ratio (Kollmer et al., 2018).

Finally, in studies comparing multiple diabetes groups (e.g., with or without DPN), disease duration is an important variable to consider, as whilst various MRI measures become deranged in diabetic neuropathy per se, some studies show a difference between healthy controls and patients with diabetes without clinically apparent neuropathy (Eaton et al., 1996; Pham et al., 2011; Wang et al., 2018; Vaeggemose et al., 2020). At present it is unclear whether these changes represent subclinical neuropathic changes—and studies demonstrating an association between MR values and nerve conduction in patients with diabetes but no DPN would support this hypothesis—or incidental changes in nerve water content related to diabetes that have no effect on nerve function. Given this uncertainty, controlling for disease duration between DPN and non-DPN groups of diabetic patients would be prudent.

On a related point, the choice of control group differs between studies in the literature. Some studies compare patients with DPN to healthy controls (Shibata et al., 1998; Felisaz et al., 2017; Wu et al., 2017; Vaeggemose et al., 2020), others compare DPN to diabetes without neuropathy (Jende et al., 2018, 2019, 2020b) and other studies have both control groups (Griffey et al., 1988; Eaton et al., 1996; Pham et al., 2011, 2015; Vaeggemose et al., 2017a,b, 2020; Wang et al., 2018; Edward et al., 2020; Groener et al., 2020; Jende et al., 2020b). Including both control groups would be preferable, as this allows to distinguish changes related to diabetes and those directly related to neuropathy. However, MRI studies are expensive and resource-intense, and sample sizes can be around 30–40 per group in well-powered studies. Therefore, of the two potential control groups, patients with diabetes without neuropathy may be the preferable choice, as these will control for confounds related to the underlying diabetic pathology including cardiovascular risk factors.

Accounting for confounding variables can be achieved in a number of ways. Firstly, groups can be “matched” for pertinent variables, most commonly done for age and gender. Alternatively, a variable can be controlled for statistically by using multiple regression or partial correlation methods. It is worth remembering for the latter approach that adding additional independent variables into a model risks compromising statistical power. Therefore, improved accounting for confounding variables by group-matching will preserve power and require smaller sample sizes.

### Sample Size and Power Calculations

Our risk of bias assessment has shown that sample size estimations and power calculations are consistently absent from studies in this this field. This is likely linked to the small number of studies having been published on this subject, with only 18 studies meeting the criteria of this review to date, most of which have been published in the past 5 years. Therefore, much of the work thus far has been relatively exploratory, and it would have been difficult until recently to estimate effect sizes. Also, analysis approaches differ substantially between studies which may impact differentially on sample size requirements and experimental power. However, there are probably sufficient data now using *T*_2_-weighted MRI, DTI, and measures of nerve cross sectional area to be able to make more informed decisions regarding expected effect sizes in the future. For example, with the approach taken by Jende et al. in analysis *T*_2_-weighted hyperintense and hypointense lesions, group sizes of around 40 seem to be required (Cohen's d = 0.62). Effect size for nerve CSA calculated from Jende et al. (2019) is slightly higher (Cohen's d = 0.71), suggesting a minimum sample size of 30 per group. However, it is noteworthy that results are inconsistent between different studies for nerve CSA, with some finding no significant difference (Jende et al., 2020b). DTI studies to date have used around 10 participants per group, which is around the minimal sample size given the effect sizes in these studies (Cohen's d = 1.24). Given that there seems to be trends for some metrics which did not consistently meet statistical significance (axial, radial and mean diffusivity), it may be that these studies were underpowered. Aiming for larger samples of 20–30 subjects per group in future studies may be beneficial.

### Anatomy Scanned, Magnetic Field Strength, and Image Resolution

Whilst early studies in this field scanned the sural nerve at the level of the ankle (Griffey et al., 1988; Koechner et al., 1995; Eaton et al., 1996; Shibata et al., 1998), most of the studies published since 2011 have focused on the sciatic nerve and the proximal extent of one of its branches (tibial nerve, common peroneal nerve) (Pham et al., 2011; Wu et al., 2017; Jende et al., 2018, 2019, 2020b; Groener et al., 2020). The sciatic nerve at the level of the thigh is significantly larger compared with more distal nerves, making it more feasible to get sufficient resolution for analysis within the limits of current magnets and pulse sequences. Also, some studies have shown a proximal-distal gradient, with a greater number of lesions at the level of the thigh than in the calf, both for lesion-load on *T*_2_-weighted imaging (Pham et al., 2015), and fractional anisotropy measures (Vaeggemose et al., 2017a,b). This gradient seems at odds to symptoms in DPN, which seem to have a more distal predominance. However, respective authors note that the multifocal involvement of the fascicles of the thigh would correspond to histopathological findings seen at this location (Dyck et al., 1986). Some studies have also investigated more distal nerves such as distal tibial and common peroneal nerves (Pham et al., 2015; Felisaz et al., 2017; Vaeggemose et al., 2017a,b, 2020; Wu et al., 2017), the sural nerve (Griffey et al., 1988; Koechner et al., 1995; Eaton et al., 1996; Shibata et al., 1998), and the median nerve in the arm (Edward et al., 2020).

In terms of magnetic field strength, all included studies used a 3 T MRI system, except for the earliest studies in the 1980's and early ‘90s (Griffey et al., 1988; Koechner et al., 1995; Eaton et al., 1996; Shibata et al., 1998) and one more recent diffusion tensor imaging study (Edward et al., 2020). This has allowed excellent spatial resolution and anatomic detail to be obtained for the relatively small structures imaged, with resolutions of around 0.3 × 0.3 mm^2^ to 0.3 × 0.5 mm^2^ in-plane, using 3–4 mm slice thickness for *T*_2_-weighted imaging (Pham et al., 2011, 2015; Vaeggemose et al., 2017a,b, 2020; Wu et al., 2017; Jende et al., 2018, 2019, 2020b). Studies using DTI have typically obtained a resolution of 1.25 × 1.25 mm^2^ to 1.36 × 1.36 mm^2^ in-plane resolution using slice thicknesses of 3–4 mm (Vaeggemose et al., 2017a,b, 2020; Wu et al., 2017; Jende et al., 2020a). One exception to the above is a study by Felisaz et al. (2017), who performed Dixon imaging in the distal tibial nerve at 3 T, using a spoiled gradient sequence with IDEAL, and by using a very restricted field of view they were able to push to a resolution of 0.117 × 0.143 × 2 mm^3^. This impressive resolution is at the limit of what is currently achievable with 3 T systems. The authors were able to segment the nerve into fascicles and epineurium, demonstrating compartment-specific changes in DPN. However, it is worth noting that all of the studies in the field so far have sacrificed resolution in the slice direction (by having greater slice thickness) to provide optimal in-plane resolution. Whilst improving SNR, this will also create partial volume effects in the slice direction, leading to some small lesions potentially being missed.

### Methods of Image Segmentation and Analysis

Analysing the MR indices described above generally requires a segmentation of nerve tissue from non-nerve tissue, so that the given index (e.g., signal intensity, *T*_2_, measures of diffusivity) can be calculated. While some of these techniques, such as DTI, use pre-existing software (generally adapted from brain imaging), which use in-built (semi)-automated segmentation, all studies focusing on *T*_2_-weighted MRI use an initial fully manual segmentation (Felisaz et al., 2017; Vaeggemose et al., 2017a,b, 2020; Jende et al., 2018, 2019, 2020b; Groener et al., 2020), generally performed by trained neuroradiologists with experience of nerve imaging. Manual segmentation is extremely time consuming and will become more so with the availability of higher resolution imaging. When multiple researchers are performing segmentation, it is also important to show intra- and inter-rater reliability, which is generally not discussed in papers to date. If the expectation is that segmenters should have substantial clinical training in neuroradiology, this is an unnecessary barrier to carrying out nerve imaging research. Therefore, adapting (semi)-automated algorithms from brain imaging research or creating custom algorithms for nerve imaging should be a research priority over the coming years.

Finally, in terms of methods of analysis the greatest variability in the literature comes from *T*_2_-weighted MRI. Whilst it is possible to calculate absolute *T*_2_ and proton density values (Pham et al., 2015; Wu et al., 2017; Vaeggemose et al., 2020), the required sequences are often more time consuming, but can give useful information about the source of *T*_2_-weighted signal intensity and shed light on the underlying pathophysiology. Another approach has been to take *T*_2_-weighted signal intensities and average them over an area of nerve to compare between groups (Pham et al., 2011), or to identify values above a cut of as “hyperintense lesions” (Pham et al., 2015). These approaches are problematic in light of subsequent data from Jende et al. (2018), demonstrating convincingly that there are areas of both hyper- and hypointense lesions, possibly reflecting different pathogenic mechanisms. Therefore, any assessment using *T*_2_-weighted MRI should assess both of these lesion types.

### Future Directions

A number of priorities for future directions are apparent from the above review of available literature. The first relates to the fact there are no published studies on HIV neuropathy, despite 18 studies to date having developed MRI biomarkers of DPN. Broadening the scope of research to other forms of neuropathy, including HIV and chemotherapy-induced neuropathy, would allow us to answer questions on how specific these biomarkers are to DPN, or whether they represent nerve damage across a range of aetiologies. There is also significant debate about the pathological underpinnings of the changes apparent in diabetic neuropathy using MRI. Comparing and contrasting MRI signatures of neuropathies of different aetiologies, with different underlying pathological processes, may help to shed light on this issue.

Similarly, longitudinal imaging will also be of great value. By comparing the same patients at multiple timepoints we will be able to track the evolution of MRI changes and relate them to changes in other biomarkers and clinical assessments. This will also be a more powerful way to study if there are objective imaging changes which occur when transitioning from absence to presence of neuropathy, potentially allowing for the development of pre-symptomatic biomarkers which predict the risk of developing clinical neuropathy. Some researchers have already begun to investigate whether MRI can detect specific changes in the nerve that relate to the development of neuropathic pain (Jende et al., 2020b). Longitudinal imaging comparing patients before and after the development of neuropathic pain, or with increasing severities of neuropathic pain, would be a powerful tool in this regard. As discussed above, the single time point design used by previous studies led to difficulties in interpreting causal relationships between blood biomarkers and MRI signatures of neuropathy, for example whether high or low serum lipids confer increased risk of developing neuropathy (Jende et al., 2019). Tracking these relationships over time in cohort studies would help clarify.

In terms of technical aspects, ultra-high field MRI (e.g., 7 T) is becoming increasingly available and will allow even greater signal-to-noise, and therefore spatial resolution, than the current body of research discussed above. Felisaz et al. (2017) have pushed the resolution at 3 T to its extreme, achieving a resolution of 0.117 × 0.147 × 2 mm^3^ using *T*_1_-weighted and Dixon imaging. At this level, the authors offer a tantalising glimpse at fascicular resolution nerve imaging with the ability to segment out structures within the nerve. Improving further on this resolution will allow more in-depth understanding of how individual nerve compartments change structurally over the course of DPN. The improved resolution available at 7 T will also be able to probe whether the increased proximal density of hyperintense and hypointense lesions shown in previous studies (e.g., Pham et al., 2015) is a true representation of the pathology in DPN, or is simply related to decreased sensitivity for picking up lesions at the smaller, distal extent of nerves when imaging at lower field strength. Finally, whilst there are methods for functional brain imaging to obtain indirect measures of activity of brain tissue, no such techniques exist yet for nerve imaging. There are numerous reasons for this, for example the clear neurovascular coupling which exists in the brain, with spatio-temporal relationships. Similar blood-flow measures are unlikely to be of value in nerve imaging. That is not, however, to say that measures will not be able to be developed which probe the ability of nerves to function, and we refer readers to the paper in this edition by Jende et al. (2021), exploring DTI as a surrogate measure of nerve function.

## Protocol and Registration

The protocol used in this systematic review was published a priori on the University of York/National Institute of Health Research (NIHR) international prospective register of systematic reviews (PROSPERO) at the following link https://www.crd.york.ac.uk/prospero/display_record.php?RecordID=167322.

In the original published protocol (Evans et al., 2020), we had aimed to search until Jan 16th 2020. However, preparation of this review was delayed by the coronavirus pandemic, and we therefore decided to extend the search to include the most up to date journal articles. Otherwise, the study was carried out as described in the original protocol.

## Data Availability Statement

The original contributions presented in the study are included in the article/supplementary material, further inquiries can be directed to the corresponding author/s.

## Author Contributions

ME, CW, AR, and DS were involved in overall structure and concept for review. Data collection/extraction and analysis by ME, CW, DH-S, AU, and KS. Manuscript writing by ME. All authors contributed to editing of manuscript.

## Funding

ME was funded by a UK National Institute of Health Research (NIHR) academic clinical fellowship (ACF). ME and AR are funded by a grant by the UK Medical Research Council (MRC) and Versus Arthritis (PAINTORM - MR/W002388/1). PL was funded by a Wellcome Trust Fellowship. DS receives grants from the Dementia Research Institute (DRI), MRC and NIHR.

## Conflict of Interest

AR undertakes consultancy and advisory board work for Imperial College Consultants- in the last 24 months this has included remunerated work for: Abide, Confo, Vertex, Pharmanovo, Lateral, Novartis, Mundipharma, Orion, Shanghai SIMR Biotech, Asahi Kasei, Toray & Theranexis. AR was the owner of share options in Spinifex Pharmaceuticals from which personal benefit accrued upon the acquisition of Spinifex by Novartis in July 2015 and from which future milestone payments may occur. AR is also named as an inventor on patents: AR, Vandevoorde S., and Lambert D. M Methods using N-2-propenylhexadecanamide and related amides to relieve pain. WO 2005/079771. Okuse K. et al. Methods of treating pain by inhibition of vgf activity EP13702262.0/ WO2013 110945. The remaining authors declare that the research was conducted in the absence of any commercial or financial relationships that could be construed as a potential conflict of interest.

## Publisher's Note

All claims expressed in this article are solely those of the authors and do not necessarily represent those of their affiliated organizations, or those of the publisher, the editors and the reviewers. Any product that may be evaluated in this article, or claim that may be made by its manufacturer, is not guaranteed or endorsed by the publisher.

## References

[B1] Behse F. Buchthal F. Carlsen F. (1977). Nerve biopsy and conduction studies in diabetic neuropathy. J. Neurol. Neurosurg. Psychiatry 40, 1072–1082. 10.1136/jnnp.40.11.1072 599355 PMC492905

[B2] Breiner A. Qrimli M. Ebadi H. Alabdali M. Lovblom L. E. Abraham A.. (2017). Peripheral nerve high-resolution ultrasound in diabetes. Muscle Nerve 55, 171–178 10.1002/mus.25223 27312883

[B3] Brownlee M. (2001). Biochemistry and molecular cell biology of diabetic complications. Nature 414, 813–820. 10.1038/414813a 11742414

[B4] Calcutt N. A. (2020). Diabetic neuropathy and neuropathic pain: a (con)fusion of pathogenic mechanisms? Pain 161, S65–S86. 10.1097/j.pain.0000000000001922 32999525 PMC7521457

[B5] Calcutt N. A. Fernyhough P. (2016). A brief introduction to the history and controversies of clinical trials in diabetic neuropathy. Int. Rev. Neurobiol. 127, 3–8. 27133141 10.1016/bs.irn.2016.03.014

[B6] Cartwright M. S. Passmore L. V. Yoon J. S. Brown M. E. Caress J. B. Walker F. O. (2008). Cross-sectional area reference values for nerve ultrasonography. Muscle Nerve 37, 566–571. 10.1002/mus.21009 18351581

[B7] Chalk C. Benstead T. J. More F. (2007). Aldose reductase inhibitors for the treatment of diabetic polyneuropathy. Cochrane Database Syst. Rev. 2010. 10.1002/14651858.CD004572.pub2 17943821 PMC8406996

[B8] Cherry C. L. Kamerman P. R. Bennett D. L. Rice A. S. (2012a). HIV-associated sensory neuropathy: Still a problem in the post-stavudine era? Future Virol. 7, 849–854. 10.2217/fvl.12.77

[B9] Cherry C. L. Wadley A. L. Kamerman P. R. (2012b). Painful HIV-associated sensory neuropathy. Pain Manag. 2, 543–552. 10.2217/pmt.12.67 24645886

[B10] Cherry C. L. Wadley A. L. Kamerman P. R. (2016). Diagnosing and treating HIV-associated sensory neuropathy: a global perspective. Pain Manag. 6, 191–199. 10.2217/pmt.15.65 26988147

[B11] Chung S. S. M. (2003). Contribution of polyol pathway to diabetes-induced oxidative stress. J. Am. Soc. Nephrol. 14, 233S–236. 10.1097/01.ASN.0000077408.15865.06 12874437

[B12] Cudlip S. A. Howe F. A. Clifton A. Schwartz M. S. Bell B. A. (2002). Magnetic resonance neurography studies of the median nerve before and after carpal tunnel decompression. J. Neurosurg. 96, 1046–1051. 10.3171/jns.2002.96.6.1046 12066905

[B13] Dyck P. J. Karnes J. L. O'Brien P. Okazaki H. Lais A. Engelstad J. (1986). The spatial distribution of fiber loss in diabetic polyneuropathy suggests ischemia. Ann. Neurol. 19, 440–449. 10.1002/ana.410190504 3717907

[B14] Eaton R. P. Quails C. Bicknell J. Sibbitt W. L. King M. K. Griffey R. H. (1996). Structure-function relationships within peripheral nerves in diabetic neuropathy: the hydration hypothesis. Diabetologia 39, 439–446. 10.1007/BF00400675 8777993

[B15] Edward R. Abdelalim A. M. Ashour A. S. Afifi L. Al-Athwari A. (2020). A study of diffusion tensor imaging of median nerve in diabetic peripheral neuropathy. Egypt. J. Neurol. Psychiatry Neurosurg. 56:42. 10.1186/s41983-020-00172-5

[B16] Evans M. C. Hohenschurz-Schmidt D. Ugwudike A. Shah N. Bangerter N. Rice A. S. C.. (2020). A systematic review of magnetic resonance imaging (MRI) studies of diabetic and HIV peripheral neuropathy. PROSPERO Int. Prospect. Regist. Syst. Rev. 10.3389/fnins.2021.727311 PMC8490874 34621152

[B17] Felisaz P. F. Maugeri G. Busi V. Vitale R. Balducci F. Gitto S.. (2017). MR micro-neurography and a segmentation protocol applied to diabetic neuropathy. Radiol. Res. Pract. 2017:2761818. 10.1155/2017/2761818 28567306 PMC5439248

[B18] Finnerup N. B. Attal N. Haroutounian S. McNicol E. Baron R. Dworkin R. H.. (2015). Pharmacotherapy for neuropathic pain in adults: a systematic review and meta-analysis. Lancet. Neurol. 14, 162–173. 10.1016/S1474-4422(14)70251-0 25575710 PMC4493167

[B19] Freeman O. J. Unwin R. D. Dowsey A. W. Begley P. Ali S. Hollywood K. A.. (2016). Metabolic dysfunction is restricted to the sciatic nerve in experimental diabetic neuropathy. Diabetes 65, 228–238. 10.2337/db15-0835 26470786

[B20] Gaist D. Jeppesen U. Andersen M. García Rodríguez L. A. Hallas J. Sindrup S. H. (2002). Statins and risk of polyneuropathy: a case-control study. Neurology 58, 1333–1337. 10.1212/WNL.58.9.1333 12011277

[B21] Gerevini S. Agosta F. Riva N. Spinelli E. G. Pagani E. Caliendo G.. (2016). Mr imaging of Brachial Plexus and limb-girdle muscles in patients with amyotrophic lateral sclerosis. Radiology 279, 553–561. 10.1148/radiol.2015150559 26583760

[B22] Griffey R. H. Eaton R. P. Sibbitt R. R. Sibbitt W. L. Bicknell J. M. (1988). Diabetic neuropathy: structural analysis of nerve hydration by magnetic resonance spectroscopy. JAMA 260, 2872–2878. 10.1001/jama.1988.03410190120034 3141635

[B23] Groener J. B. Jende J. M. E. Kurz F. T. Kender Z. Treede R. D. Schuh-Hofer S.. (2020). Understanding diabetic neuropathy—from subclinical nerve lesions to severe nerve fiber deficits: a cross-sectional study in patients with type 2 diabetes and healthy control subjects. Diabetes 69, 436–447. 10.2337/db19-0197 31826867

[B24] Hebert P. R. Gaziano J. M. Chan K. S. Hennekens C. H. (1997). Cholesterol lowering with statin drugs, risk of stroke, and total mortality. An overview of randomized trials. JAMA 278, 313–321. 10.1001/jama.1997.03550040069040 9228438

[B25] International_Diabetes_Federation (2015). IDF Diabetes Atlas. Available online at: http://www.diabetesatlas.org (accessed June 6, 2021).

[B26] Jende J. M. E. Groener J. B. Kender Z. Hahn A. Morgenstern J. Heiland S.. (2020a). Troponin T parallels structural nerve damage in type 2 diabetes: a cross-sectional study using magnetic resonance neurography. Diabetes 69, 713–723. 10.2337/db19-1094 31974140

[B27] Jende J. M. E. Groener J. B. Kender Z. Rother C. Hahn A. Hilgenfeld T.. (2020b). Structural nerve remodeling at 3-T MR neurography differs between painful and painless diabetic polyneuropathy in type 1 or 2 diabetes. Radiology 294, 405–414. 10.1148/radiol.2019191347 31891321

[B28] Jende J. M. E. Groener J. B. Oikonomou D. Heiland S. Kopf S. Pham M.. (2018). Diabetic neuropathy differs between type 1 and type 2 diabetes: insights from magnetic resonance neurography. Ann. Neurol. 83, 588–598. 10.1002/ana.25182 29443416

[B29] Jende J. M. E. Groener J. B. Rother C. Kender Z. Hahn A. Hilgenfeld T.. (2019). Association of serum cholesterol levels with peripheral nerve damage in patients with type 2 diabetes. JAMA Netw. Open 2:e194798. 10.1001/jamanetworkopen.2019.4798 31150078 PMC6547108

[B30] Jende J. M. E. Kender Z. Mooshage C. Groener J. B. Alvarez-Ramos L. Kollmer J.. (2021). Diffusion tensor imaging of the sciatic nerve as a surrogate marker for nerve functionality of the upper and lower limb in patients with diabetes and prediabetes. Front. Neurosci. 15:642589. 10.3389/fnins.2021.642589 33746707 PMC7966816

[B31] Jende J. M. E. Kender Z. Rother C. Alvarez-Ramos L. Groener J. B. Pham M.. (2020c). Diabetic polyneuropathy is associated with pathomorphological changes in human dorsal root ganglia: a study using 3T MR neurography. Front. Neurosci. 14:570744. 10.3389/fnins.2020.570744 33100960 PMC7546893

[B32] Kamerman P. R. Wadley A. L. Cherry C. L. (2012). HIV-associated sensory neuropathy: risk factors and genetics. Curr. Pain Headache Rep. 16, 226–236. 10.1007/s11916-012-0257-z 22367397

[B33] Koechner D. Petropoulos H. Eaton R. P. Hart B. L. Brooks W. M. (1995). Segmentation of small structures in MR images: semiautomated tissue hydration measurement. J. Magn. Reson. Imaging 5, 347–351. 10.1002/jmri.1880050320 7633113

[B34] Kollmer J. Kästel T. Jende J. M. E. Bendszus M. Heiland S. (2018). Magnetization transfer ratio in peripheral nerve tissue: does it depend on age or location? Invest. Radiol. 53, 397–402. 10.1097/RLI.0000000000000455 29470194

[B35] Kronlage M. Bäumer P. Pitarokoili K. Schwarz D. Schwehr V. Godel T.. (2017). Large coverage MR neurography in CIDP: diagnostic accuracy and electrophysiological correlation. J. Neurol. 264, 1434–1443. 10.1007/s00415-017-8543-7 28620719

[B36] Kronlage M. Schwehr V. Schwarz D. Godel T. Heiland S. Bendszus M.. (2019). Magnetic resonance neurography: normal values and demographic determinants of nerve caliber and T2 relaxometry in 60 healthy individuals. Clin. Neuroradiol. 29, 19–26. 10.1007/s00062-017-0633-5 29030674

[B37] Kronlage M. Schwehr V. Schwarz D. Godel T. Uhlmann L. Heiland S.. (2018). Peripheral nerve diffusion tensor imaging (DTI): normal values and demographic determinants in a cohort of 60 healthy individuals. Eur. Radiol. 28, 1801–1808. 10.1007/s00330-017-5134-z 29230526

[B38] Lauria G. Hsieh S. T. Johansson O. Kennedy W. R. Leger J. M. Mellgren S. I.. (2010). European Federation of Neurological Societies/Peripheral Nerve Society guideline on the use of skin biopsy in the diagnosis of small fiber neuropathy. Report of a joint task force of the European Federation of Neurological Societies and the Peripheral. Ner. J. Peripher. Nerv. Syst. 17, 903-12, e44–e49. 10.1111/j.1468-1331.2010.03023.x 20626771

[B39] Lee C. Liu Q.-H. Tomkowicz B. Yi Y. Freedman B. D. Collman R. G. (2003). Macrophage activation through CCR5- and CXCR4-mediated gp120-elicited signaling pathways. J. Leukoc. Biol. 74, 676–682. 10.1189/jlb.0503206 12960231

[B40] Lee D. Dauphinée D. M. (2005). Morphological and functional changes in the diabetic peripheral nerve: using diagnostic ultrasound and neurosensory testing to select candidates for nerve decompression. J. Am. Podiatr. Med. Assoc. 95, 433–437. 10.7547/0950433 16166459

[B41] Leinninger G. M. Vincent A. M. Feldman E. L. (2004). The role of growth factors in diabetic peripheral neuropathy. J. Peripher. Nerv. Syst. 9, 26–53. 10.1111/j.1085-9489.2004.09105.x 14871451

[B42] Moss P. J. Huang W. Dawes J. Okuse K. McMahon S. B. Rice A. S. C. (2015). Macrophage–sensory neuronal interaction in HIV-1 gp120-induced neurotoxicity. Br. J. Anaesth. 114, 499–508. 10.1093/bja/aeu311 25227937 PMC4332570

[B43] Novak P. Pimentel D. A. Sundar B. Moonis M. Qin L. Novak V. (2015). Association of statins with sensory and autonomic ganglionopathy. Front. Aging Neurosci. 7:191. 10.3389/fnagi.2015.00191 26500548 PMC4595790

[B44] Page M. J. McKenzie J. E. Bossuyt P. M. Boutron I. Hoffmann T. C. Mulrow C. D.. (2021). The PRISMA 2020 statement: an updated guideline for reporting systematic reviews. BMJ 372:n71. 10.1136/bmj.n71 33782057 PMC8005924

[B45] Pham M. Oikonomou D. Baumer P. Bierhaus A. Heiland S. Humpert P. M.. (2011). Proximal neuropathic lesions in distal symmetric diabetic polyneuropathy findings of high-resolution magnetic resonance neurography. Diabetes Care 34, 721–723. 10.2337/dc10-1491 21266652 PMC3041214

[B46] Pham M. Oikonomou D. Hornung B. Weiler M. Heiland S. Bäumer P.. (2015). Magnetic resonance neurography detects diabetic neuropathy early and with proximal predominance. Ann. Neurol. 78, 939–948. 10.1002/ana.24524 26381658 PMC5132066

[B47] Riazi S. Bril V. Perkins B. A. Abbas S. Chan V. W. S. Ngo M.. (2012). Can ultrasound of the tibial nerve detect diabetic peripheral neuropathy?: a cross-sectional study. Diabetes Care 35, 2575–2579. 10.2337/dc12-0739 23033242 PMC3507587

[B48] Schmid A. B. Campbell J. Hurley S. A. Jbabdi S. Andersson J. L. Jenkinson M.. (2018). Feasibility of diffusion tensor and morphologic imaging of peripheral nerves at ultra-high field strength. Invest. Radiol. 53, 705–713. 10.1097/RLI.0000000000000492 29979328 PMC6221405

[B49] Scott W. (2019). The psychosocial context of chronic pain in people living with HIV. PAIN Rep. 4:e721. 10.1097/PR9.0000000000000721 31334439 PMC6455682

[B50] Scott W. Arkuter C. Kioskli K. Kemp H. McCracken L. M. Rice A. S. C.. (2018). Psychosocial factors associated with persistent pain in people with HIV: a systematic review with meta-analysis. Pain 159, 2461–2476. 10.1097/j.pain.0000000000001369 30130299 PMC6250281

[B51] Seok H. Y. Jang J. H. Won S. J. Yoon J. S. Park K. S. Kim B. J. (2014). Cross-sectional area reference values of nerves in the lower extremities using ultrasonography. Muscle Nerve 50, 564–570. 10.1002/mus.24209 24639103

[B52] Shibata T. Suzuki E. Yasuda K. Yasuda K. (1998). Effects of aldose reductase inhibitor and prostaglandin I2 analogue on diabetic neuropathy - Clinical study with magnetic resonance imaging. J. Japan Diabetes Soc. 41, 423–431. 10.11213/tonyobyo1958.41.423

[B53] Smith A. G. Howard J. R. Kroll R. Ramachandran P. Hauer P. Singleton J. R.. (2005). The reliability of skin biopsy with measurement of intraepidermal nerve fiber density. J. Neurol. Sci. 228, 65–69. 10.1016/j.jns.2004.09.032 15607212

[B54] Tesfaye S. (2011). Recent advances in the management of diabetic distal symmetrical polyneuropathy. J. Diabetes Investig. 2, 33–42. 10.1111/j.2040-1124.2010.00083.x 24843458 PMC4008012

[B55] Tesfaye S. Stevens L. K. Stephenson J. M. Fuller J. H. Plater M. Ionescu-Tirgoviste C.. (1996). Prevalence of diabetic peripheral neuropathy and its relation to glycaemic control and potential risk factors: the EURODIAB IDDM complications study. Diabetologia 39, 1377–1384. 10.1007/s001250050586 8933008

[B56] Tievsky A. L. Ptak T. Farkas J. (1999). Investigation of apparent diffusion coefficient and diffusion tensor anisotrophy in acute and chronic multiple sclerosis lesions. AJNR. Am. J. Neuroradiol. 20, 1491–1499. 10512236 PMC7657750

[B57] Vaeggemose M. Haakma W. Pham M. Ringgaard S. Tankisi H. Ejskjaer N.. (2020). Diffusion tensor imaging MR Neurography detects polyneuropathy in type 2 diabetes. J. Diabetes Complications 34:107439. 10.1016/j.jdiacomp.2019.107439 31672457

[B58] Vaeggemose M. Pham M. Ringgaard S. Tankisi H. Ejskjaer N. Heiland S.. (2017a). Diffusion tensor imaging MR neurography for the detection of polyneuropathy in type 1 diabetes. J. Magn. Reson. Imaging 45, 1125–1134. 10.1002/jmri.25415 27472827

[B59] Vaeggemose M. Pham M. Ringgaard S. Tankisi H. Ejskjaer N. Heiland S.. (2017b). Magnetic resonance neurography visualizes abnormalities in sciatic and tibial nerves in patients with type 1 diabetes and neuropathy. Diabetes 66, 1779–1788. 10.2337/db16-1049 28432188

[B60] Van Hecke O. Austin S. K. Khan R. A. Smith B. H. Torrance N. (2014). Neuropathic pain in the general population: a systematic review of epidemiological studies. Pain 155, 654–662. 10.1016/j.pain.2013.11.013 24291734

[B61] Wang D. Wang C. Duan X. Yang Z. Bai Z. Hu H.. (2018). MR T2 value of the tibial nerve can be used as a potential non-invasive and quantitative biomarker for the diagnosis of diabetic peripheral neuropathy. Eur. Radiol. 28, 1234–1241. 10.1007/s00330-017-5043-1 29038932

[B62] Wodarski R. Bagdas D. Paris J. J. Pheby T. Toma W. Xu R.. (2018). Reduced intraepidermal nerve fibre density, glial activation, and sensory changes in HIV type-1 Tat-expressing female mice. PAIN Rep. 3:e654. 10.1097/PR9.0000000000000654 29922746 PMC5999412

[B63] Won J. C. Kwon H. S. Kim C. H. Lee J. H. Park T. S. Ko K. S.. (2012). Prevalence and clinical characteristics of diabetic peripheral neuropathy in hospital patients with Type2 diabetes in Korea. Diabet. Med. 29, e290–e296. 10.1111/j.1464-5491.2012.03697.x 22519862

[B64] Wu C. Wang G. Zhao Y. Hao W. Zhao L. Zhang X.. (2017). Assessment of tibial and common peroneal nerves in diabetic peripheral neuropathy by diffusion tensor imaging: a case control study. Eur. Radiol. 27, 3523–3531. 10.1007/s00330-016-4698-3 28004159

[B65] Xu F. Zhao L. H. Su J. Bin Chen T. Wang X. Q. Chen J. F.. (2014). The relationship between glycemic variability and diabetic peripheral neuropathy in type 2 diabetes with well-controlled HbA1c. Diabetol. Metab. Syndr. 6:139. 10.1186/1758-5996-6-139 25530811 PMC4272789

